# Different Standards: Observing Variation in Citizens’ Respect-Based Norms for Mediated Political Communication

**DOI:** 10.1093/poq/nfaf001

**Published:** 2025-05-20

**Authors:** Emma Turkenburg, Ine Goovaerts, Sofie Marien

**Affiliations:** Assistant Professor, Strategic Communication Group, Wageningen University & Research, Wageningen, The Netherlands; Postdoctoral Researcher, Media Movement, and Politics (M^2^P), University of Antwerp, Antwerp, Belgium; Associate Professor, Centre for Political Research, KU Leuven, Leuven, Belgium

## Abstract

Incivility, oversimplification, lying, inaccessible language: there is widespread concern and controversy about the disrespectful ways politicians communicate. The reasoning underlying these worries is that such communication violates widely shared communicative norms, and that exposure to it may lead to adverse consequences in the wider public. However, widespread support for respect-based norms among citizens is generally presupposed, and little is known about the extent to which norm support matters in how people react when witnessing disrespectful politicians. Using Belgian survey data (N = 2,030), we investigate whether citizens differ in the degree to which they support different respect-based norms for mediated elite communication, and whether differing levels of norm support moderate the relationship between perceived norm violations and several political outcomes (affect toward politicians; political trust; talking about politics; political information seeking). The results reveal substantial variation in norm support across the population, with differences based on sociodemographic characteristics (e.g., education level) and political attitudes (cynical, populist, polarized attitudes). This variation, moreover, matters. While depending on the outcome and norms we study, several findings show that citizens supporting respect-based norms react more negatively when perceiving norm violations more frequently, as compared to citizens caring less about these norms. Yet, whether and in what way this moderating effect occurs can differ for different types of disrespect. As such, besides showing that respectful communication is not equally important to everyone and that not everyone reacts to norm breaking in the same way, this study also underlines that not all shades of disrespect should be tarred with the same brush.

There is a dazzling array of critiques about politicians’ communication today, including concerns about their use of incivility, dishonesty, oversimplification, and inaccessible language. These critiques are usually based on the assumed violation of some sort of communicative *norms*, many of which constitute broad manifestations of showing *respect* in political communication. Here, showing respect is linked to being respectful not only toward one’s conversation partner (e.g., political opponent), but also toward one’s audience (e.g., TV audience at home; Turkenburg 2022; Stryker et al. 2024). In effect, civil language, accessibly justified standpoints, and provision of truthful information all signal respect to message receivers. These normative standards were previously mainly advanced by academics or derived from theory in a top-down manner. Today, various bottom-up approaches, especially in incivility research, show that violations of theoretical respect-based norms are often perceived as disrespectful by citizens, too (e.g., Bormann et al. 2022; Bentivegna and Rega 2022; Stryker et al. 2021, 2024).

Yet, perceiving a communicative act as (dis)respectful is not necessarily the same as (dis)approving of it. To date, we have surprisingly little empirical knowledge about the *normative expectations* different citizens hold toward politicians’ communication, and how potential differences in norm support across citizens influence reactions to norm violations. Existing work testing effects of norm-violating communication still often assumes general agreement with these norms within society (Szabó, Kmetty, and Molnár 2021; Clementson, Zhao, and Park 2023). But is this actually the case? May citizens not differ substantially in their support for respect-based norms? Starting from this observation, this study investigates, first, whether norm support varies across citizens with different sociodemographic characteristics (gender, age, education) and political attitudes (cynical, polarized, populist attitudes). Second, we ask whether citizens with higher norm support react differently to perceived norm violations than citizens with lower norm support, and study this relationship for four political outcomes: affect toward politicians, political trust, talking about politics, and political information seeking.

Addressing these questions, we use novel Belgian survey data and focus on politicians’ communication in mediated contexts (e.g., talk shows, TV debates) because these are key venues for receiving political information. Specifying this is important, as norms can differ across contexts (e.g., Otto, Lecheler, and Schuck 2020). The findings overall reveal that, while most people perceive politicians not to play by the rules, not everyone attaches importance to these rules to the same extent. As a result, not everyone may react to rule breaking in the same way.

## Respect-Based Norms for Political Communication

Social norms, as understood here, concern behavior considered appropriate in a social group. Norms are often shaped at least partly by the judgements and expectations of others in one’s social group. Yet, people always hold individual interpretations—perceived individual norms—of collective norms (Ajzen and Fishbein 1980; Cialdini, Kallgren, and Reno 1991; Lapinski and Rimal 2005; Rimal and Lapinski 2015). Viewing communication as a form of social behavior, we view norms here as an individual’s expectations of how politicians ought to communicate appropriately in a given social situation (paraphrasing Bormann et al. 2022, p. 10), thus reflecting an *ideal* one has. Such ideals are also termed *injunctive* norms, and are different from *descriptive* norms, which relate to one’s perception of how politicians *actually* behave in certain contexts (Kallgren, Reno, and Cialdini 2000; Muddiman, Flores, and Boyce 2022). Building on Muddiman et al. (2022), we include both types. First, we expect that norm support (injunctively) differs across citizens. Second, we expect that those with stronger norm support react differently to norm violations (i.e., politicians’ actual communication; descriptively) than those with lower norm support.

To test these expectations, we focus on *respect-based* communicative norms. Drawing mainly on the incivility literature, norm-adhering, respectful communication is understood here as politicians’ use of civil, substantive, truthful communication that is also accessible to the wider public. Not interrupting or rudely discrediting opponents, providing truthful information, and explaining standpoints substantively and accessibly all feature in and across different incivility studies. These norms are not only derived in a top-down way: violations of these norms are judged as disrespectful by citizens as well. In effect, prior studies show that different norms can be subsumed under one overarching but multidimensional construct of perceived disrespect, or political incivility in a broader sense. Although the precise (in)civility subdimensions in these studies sometimes differ—depending on the studied context and norms—the main observation is that these specific norms all relate to the broader concept of (dis)respect (e.g., Stryker, Conway, and Danielson 2016; Muddiman 2017; Bentivegna and Rega 2022; Bormann et al. 2022; Stryker et al. 2021, 2024). As Stryker et al. (2024) show, the “common core” of these different communicative norms is (dis)respect, “whether for one's political interlocutors, one’s political audience, and/or the democratic polity itself” (p. 19).

In the studied context of mediated political communication, we are indeed not only talking about mutual respect between politicians, but at least as much about respect toward the audience as a serious partner in communication (Turkenburg 2022). When politicians appear in the media, they do not only discuss politics with their political opponents, they also (and often primarily) communicate toward the audience at home. In effect, norm violations such as interrupting others do not only display disrespect toward the opponent; they also hamper equal communication and prevent necessary information from reaching the audience (Popan et al. 2019).^1^

Based on this, we include various norms that can all be conceptually classified and grounded in this broader ideal of respectful communication toward one’s interlocutor and audience. Although we mainly build on political incivility literature, many respect-based norms we identify also connect to other fields,^2^ such as deliberative democratic theory (e.g., Habermas 1984; Steenbergen et al. 2003; Bächtiger et al. 2018), argumentation theory (e.g., Toulmin 1958; Van Eemeren and Grootendorst 2004), politeness theory (e.g., Brown and Levinson 1987), and the mis- and disinformation literature (e.g., Hameleers and Minihold 2022; Hameleers, van der Meer, and Vliegenthart 2022).

In the (in)civility literature, frequently encountered norms include politeness (not being rude or insulting), allowing others to speak (not interrupting), and engaging with, rather than derogating, opponents’ viewpoints (Stryker, Conway, and Danielson 2016; Muddiman 2017; Bormann et al. 2022).^3^ Adhering to these norms can be seen as “a way of showing mutual respect” (Mutz 2015, p. 22). Civility also features prominently in the deliberative democracy scholarship as one of the broader manifestations of respect (Gutmann and Thompson 1996).

Furthermore, disrespect can manifest through the use of unsophisticated communication. Sophisticated speech entails the provision of justified, complete, truthful, sufficiently nuanced information. This is important from a normative democratic perspective, as it allows people to make well-reasoned decisions, and because politicians are expected to explain why they take certain decisions (Pitkin 1967; Chambers 2010). Oversimplifying, lying, or not providing justifications deprives an audience of input and tools to form their opinion (Bormann 2022; Stryker et al. 2021). Sophisticated communication signals that the conversation partner and audience are respected enough to give them all necessary and relevant information (Bischof and Senninger 2022; Bormann et al. 2022).

Importantly, sophisticated communication does not equate complex communication. The provision of complete, well-justified information in such a way that it is actually understandable and accessible is another important feature in communication (Holtz-Bacha 2004). It can be seen as disrespectful to disregard the understanding of communicative partners by using hedging and ambiguous language, making communication complicated, technocratic, and dense (Bormann 2022; Bormann et al. 2022). Not tailoring the delivery of information to an audience can make communication difficult to receive, access, and understand, especially for minority groups in society that are not part of the elite (Young 2000; Bächtiger et al. 2018).

Overall, these communicative norms appear in both theoretical top-down frameworks and in studies approaching (in)civility as a perceptual construct. Hence, theoretical violations of respect-based norms are also often judged by citizens as disrespectful. Still, recognizing something as (dis)respectful is not the same as (dis)approving of it. Therefore, the question remains whether support for respect-based norms is as widely shared as is often assumed.

## Adherence to Respect-Based Norms Across the Population

Despite increasing attention to citizens’ differential reactions to norm-violating political communication, the prominent idea is that, regardless of context or venue, the norm of communicating respectfully is widely shared (Szabó, Kmetty, and Molnár 2021; Clementson, Zhao, and Park 2023). For instance, Mutz (2015, pp. 23, 31) concludes that civility norms are similar for discussions among citizens and politicians, and for face-to-face interactions and televised debates. Accordingly, it is generally argued that incivility violates citizens’ norms, and as such negatively affects the public’s outlook on politics (Mutz 2015; Muddiman, Flores, and Boyce 2022). While many key findings indeed show main effects of politicians’ use of disrespectful communication, such as lowering political trust, or heightening affective polarization (Skytte 2021; Van’t Riet and Van Stekelenburg 2022),^4^ studies also increasingly show that effects differ across people, depending for instance on citizens’ personality traits or political attitudes (Mutz and Reeves 2005; Amsalem 2019; Muddiman 2019; Sydnor 2019; Goovaerts and Marien 2020; Guess et al. 2020; Kenski, Coe, and Rains 2020; Otto, Lecheler, and Schuck 2020; Nai and Maier 2021; Vargiu and Nai 2022).

One main explanation for this conditionality may be that certain people care less (or more) about respect in politics than others. Such potential differences have hitherto largely been overlooked, yet from social norms literature more generally, we already know that perceived individual norms can differ across citizens (e.g., Kallgren, Reno, and Cialdini 2000). How does this apply to the context of mediated political communication? While we can build on some first studies that started looking into the degree to which different citizens perceive or rate (theoretical) norm violations as uncivil or disrespectful (e.g., Kenski, Coe, and Rains 2019, 2020; Conway and Stryker 2021), research more specifically looking into citizen *support* for respect-based norms across the population is still scant. Some insights drawn from focus groups do indicate specific expectations held by citizens for politicians’ mediated communication such as in televised debates, but do not yet provide clear evidence of widely shared, or variations in, norm support (Coleman and Moss 2016).

To shed more light on individual variation in norm support, we first focus on sociodemographics. Existing and related work presents mixed findings. Jennstål et al. (2021) studied support for different communicative norms in interpersonal, deliberative settings. They show that citizens generally share deliberative norms—which somewhat links to our concept of respect-based norms—for inter-citizen political interactions. Yet, they also point to individual-level variations. No straightforward relation was found between age and support for deliberative conversational norms, but higher-educated respondents showed stronger support and female respondents held lower levels of overall support for deliberative norms. We do, however, not know whether similar standards and variations apply to the mediated communication of politicians, a more antagonistic genre. Studying perceptions of everyday incivility in the United States, Kenski et al. (2019, 2020) demonstrate that women are more likely to consider certain expressions to be uncivil, compared to men. However, they find hardly any variation for education level. Mölders and Van Quaquebeke (2017), contrastingly, find that attitudes toward political disrespect correlate with age (the older, the more accepting), but not with gender. This is also partly supported by Conway and Stryker (2021), yet it depends on the norm violation they study. While women and younger people find name-calling less acceptable, results turn insignificant for deception. To gain more insight into these nonconclusive findings, we ask the following research question:

**RQ1**: How do sociodemographics (age, gender, education level) influence levels of support for respect-based norms in mass-mediated settings?

Next, we expect variation related to citizens’ political attitudes, namely political cynicism, populist, and polarized attitudes. Previous work reports that politically cynical citizens are persuaded more by communication that violates respect-based norms than less politically cynical people, and that they are also more trusting of politicians using such communication (Goovaerts and Marien 2020). The explanation given for this finding is that highly cynical citizens may care less about such communicative norms. Similarly, effects of the communication of populist politicians, which is generally more uncivil and unsophisticated (Wyss, Beste, and Bächtiger 2015; Marien, Goovaerts, and Elstub 2020), are more persuasive for politically cynical citizens (Bos et al. 2013). Relatedly, in recent decades we have seen rising success of populist politicians and their populist communication style (Moffitt 2016; Rooduijn et al. 2019). Citizens with stronger populist attitudes have been shown to be persuaded more by norm-violating communication, to be less supportive of general democratic norms, and more intolerant of opposing views, implying they may care less about communicative norms (Bos, van der Brug, and de Vreese 2013; Plescia and Eberl 2021; Bos, Wichgers, and van Spanje 2023; Vargiu, Nai, and Valli 2024). In a similar vein, affective polarization has been connected to general norm degeneration, in line with research showing that polarization causes less willingness to collaborate and compromise with the other side (Wolf, Strachan, and Shea 2012; Oswald and Robertson 2020). Hence, the more affectively polarized, the less one may care about respectful political communication. Based on all this, we hypothesize:

**H1a**: The higher one’s level of political cynicism, the lower their support for respect-based norms.

**H1b**: The higher one’s level of populist attitudes, the lower their support for respect-based norms.

**H1c**: The higher one’s level of polarized attitudes, the lower their support for respect-based norms.

## Linking Norm Support to Political Attitudes and Engagement

Variations in norm support could prove important when studying effects of citizen exposure to norm violations. Disrespectful communication generally spurs worries because of its potential detrimental consequences: it can, for instance, lower perceived democratic legitimacy and lead citizens to tune out from politics (Lau and Redlawsk 2001; Mutz 2015). Yet, drawing on previous conditional effects findings (e.g., Amsalem 2019; Vargiu and Nai 2022), we posit that for these effects to happen, the person exposed to a norm violation also needs to adhere to said norm. Building on Muddiman et al. (2022), we argue that when perceived descriptive norms are violated (i.e., politicians *actually* communicating disrespectfully), citizens may react differently to it, depending on their level of support for respect-based injunctive norms (i.e., how respectfully citizens *want* politicians to communicate).

Indeed, Mölders and Van Quaquebeke (2017) already found that effects of politicians’ norm-violating communication are dependent on norm support in individuals. They show that citizens who care less about respectful communication in politics display more positive judgements of disrespectful politicians and also (intend to) vote for them more, as compared to citizens caring more about respectful politics. In a similar vein, Fridkin and Kenney (2011) study the role of individual differences in tolerance for negative campaigning. Their findings show that respondents who have lower tolerance for negative campaigning judge candidates using negative campaign commercials more harshly. This result is corroborated by Nai and Maier (2021), who demonstrate that low tolerance for negativity worsens people’s perceptions of candidates who use negative attacks.

Building on these findings and focusing on a broad array of respect-based norms for mediated communication, we explore how variation in norm support relates to four political outcomes: affective warmth toward politicians, political trust, talking about politics, and seeking out political information. These are all important attitudes and forms of political engagement, playing a crucial role for democracy to function well (e.g., Delli Carpini and Keeter 1996; Norris 1999). We build on previous findings showing that norm-violating communication influences these outcomes but that effects vary across individuals (e.g., Mutz and Reeves 2005; Bos, van der Brug, and de Vreese 2013; Goovaerts and Marien 2020; Otto, Lecheler, and Schuck 2020). Hence, citizens who adhere less strongly to respect-based norms are expected to react less strongly to communication that deviates from these norms. In other words, we expect norm support to operate as moderator in these relationships:

**H2**: The relation between perceived occurrence of disrespect and (a) affect toward politicians, (b) political trust, (c) political talk, and (d) information seeking is more negative among individuals with stronger support for respect-based norms.

## Method

We use cross-sectional Belgian survey data, collected by the Belgian EOS consortium RepResent,^5^ through Kantar TNS’s online panel (May 20–June 14, 2021; N_completed_ = 2,030). From Kantar TNS’s Belgian respondent pool (based on self-registration), a stratified random sample of respondents is invited, with quotas specified for age, gender, and education to match the Belgian population (see Supplementary Material section B for population percentages and full descriptives). The final sample consists of 47.6 percent male respondents and 51.9 percent female respondents, and the mean age is 48. Some 22 percent of respondents finished primary education but have no or unfinished secondary education (lower education). Another 35 percent fully completed their secondary education (intermediate education), and 43 percent completed higher education (higher professional education or university). French-speaking respondents (Wallonia, 50 percent) received the survey in French, Dutch-speaking respondents (Flanders, 50 percent) in Dutch.

This single-country case allows us to dive into variations in norm support within a society. While this does not allow generalization across borders, results can be indicative of patterns in countries similar to Belgium (democratic corporatist country, history of consensus politics, strong public broadcaster, generally impartial journalistic practices; Hallin and Mancini 2004; Deschouwer 2012; Brüggemann et al. 2014)

To measure *norm support*, respondents indicated their agreement with statements about how they want politicians to communicate in mediated political settings, for nine different manifestations of respect-based norms. Most statements were phrased *away* from the norm, formulated to limit social desirability and coded such that higher scores reflect stronger norm support. *Perceived disrespect* was measured in a similar fashion, asking respondents to evaluate today’s mediated political discussions for each norm. Item wordings are presented in table 1 and Supplementary Material section A. Supplementary Material section B includes further descriptives and a correlation matrix showing the (low) correlations between both concepts, indicating they are different constructs.

**Table 1.** Norm support and perceived disrespect items.

Norm manifestation	Norm support items	Perceived disrespect items
	In a political discussion…	Today, I see that in political discussions…
1. Truthful	Politicians do not always have to tell the truth, they are allowed to speculate.	Politicians do not tell the truth, there is frequent speculation.
2. Politeness	Politicians do not always have to be polite, a firm lashing out is allowed.	Politicians are not polite, they often lash out at each other.
3. No interruptions	Politicians do not have to discuss everything at length, one-liners and slogans are part of the game.	Politicians often use one-liners and slogans.
4. Engaging with other standpoints	Politicians should be careful with difficult language, they should always communicate in a comprehensible way that is understandable to everyone.	Politicians do not communicate in a way that is understandable for everyone.
5. To-the-point	Politicians do not always have to be “to the point” and concrete, it is okay if they digress.	Politicians are not “to the point” and concrete, but often digress.
6. Understandable	Politicians do not always have to respond to the views of others, they should mainly communicate their own ideas.	Politicians do not address the views of others and they mainly communicate their own ideas.
7. No simplifications	Politicians should not simplify matters, they can go deeper into more difficult topics.	Politicians simplify things and do not go deep into more difficult topics.
8. Justified	Politicians do not always have to let others finish when speaking; interrupting each other is part of the job.	Politicians do not let others finish speaking and they often interrupt each other.
9. No one-liners	Politicians should primarily communicate what they stand for, justification and argumentation for their positions are not always necessary.	Politicians do not give justification and argumentation for their positions but only say what they stand for.

*Note:* Agreement rated on a 1 (completely disagree) to 7 (completely agree) scale with *don't know* option. Cronbach’s alpha for norm support (7 items) = 0.802; Cronbach’s alpha for perceived disrespect (7 items) = 0.885.

As previous studies have shown that disrespect is an overarching yet multidimensional construct, we explored the existence of distinct underlying clusters of respect-based norms in our data through exploratory factor analyses (Supplementary Material section C). At first, two factors were extracted from the *norm support* items, with the sixth and seventh norm manifestation (communicating understandably and not using simplifications) loading on a different factor than the other seven norms. The seven norms loaded substantially on factor 1, and demonstrated strong internal consistency (Cronbach’s alpha = 0.802). This was not the case for the two items in factor 2 (Spearman-Brown coefficient = 0.527). Upon further inspection, the different nature of the sixth and seventh items may be caused by their formulation which is, in contrast to the other items, not phrased away from the norm. This notion was furthermore strengthened by the fact that for *perceived disrespect*, all items (phrased in the same direction) load on one singular factor, and no separate clusters were detected. Hence, we opted to generate the composite measures of norm support (M = 4.529; SD = 1.281) and perceived disrespect (M = 5.346; SD = 1.143) without the sixth and seventh items. These composite variables are used for our main analyses, but all analyses are also conducted for each separate norm (Supplementary Material section F).

To assess variation in norm support (RQ1, H1a–c), we included participants’ sociodemographics—*gender*, *age*, *education level—*and political attitudes—*political cynicism* (Goovaerts and Marien 2020), *populist attitudes* (e.g., Akkerman, Mudde, and Zaslove 2014), and *affectively polarized attitudes* (table 2). Affective polarization is usually measured by gauging citizens’ feelings toward (supporters of) different political parties. Because we are interested in affectively polarized attitudes in a more general sense, we use a battery with statements tapping into respondents’ tendency to avoid and hold negative and moralized feelings toward those with different opinions (Hetherington and Rudolph 2015; Dekker and Den Ridder 2019; McCoy and Somer 2019).

**Table 2.** Measurement overview.

Variable	Measurement
Cynicism	Agreement with statements [1–7]
Cronbach’s alpha = 0.833	Politicians deliberately promise more than they can deliver.
	Politicians mainly act out of self-interest.
	Politicians do not understand what matters to the people.
Populist attitudes	Agreement with statements [1–7]
Cronbach’s alpha = 0.838	Politicians in parliament must follow the will of the people.
	Politicians talk too much and act too little.
	The most important political decisions should be made by the people and not by politicians.
	I would rather be represented by an ordinary citizen than by a professional politician.
Affectively polarized attitudes	Agreement with statements [1–7]
Cronbach’s alpha = 0.756	There are people I have come to dislike for their views.
	I tend to avoid some people because of their opinions.
	You can tell if someone is good or bad based on their political affiliations.
Affect towards politicians	Warm/cold feelings about politicians in general on a thermometer scale [0–100 degrees]
Political trust	Trust in different institutions [0–10]
Cronbach’s alpha = 0.959	Political parties
	Federal parliament
	Politicians
	Federal government
Political talk	Frequency of discussing politics with others (friends, family, colleagues) [multiple choice]
	Never/Less than once a month/Once a month/Several times a month/Once a week/Several times a week/Daily
Political information seeking	Agreement with statements [1–7]
Cronbach’s alpha = 0.770	I closely follow what is going on in politics.
	Political programs in the media do not interest me. (rev)
	When I see politicians debating, I tune out. (rev)
	I deliberately search for political news programs.

To test whether citizens’ level of norm support moderates the relationship between perceptions of norm violations and political outcomes (H2), we assess citizens’ attitudes toward politics with a measure of *affective warmth toward politicians in general* (e.g., Gordon and Miller 2004) and a *political trust* battery. We moreover included measures related to engagement with politics. Building on Cho and Choy’s (2011) items related to everyday communication activities, we asked how often respondents *talk about politics* with others, and how much they engage with or *seek out political information* (table 2; Supplementary Material sections A and B).

For our main analyses, we use OLS regressions and report unweighted complete-case analysis (listwise deletion), whereby don’t know answers are treated as missing. In all analyses, we control for respondents’ region of residence and ideology (left-right self-placement). As robustness checks, the analyses were rerun including extra control variables and for all norm manifestations separately (Supplementary Material sections E and F).

## Results

**Figure 1.**
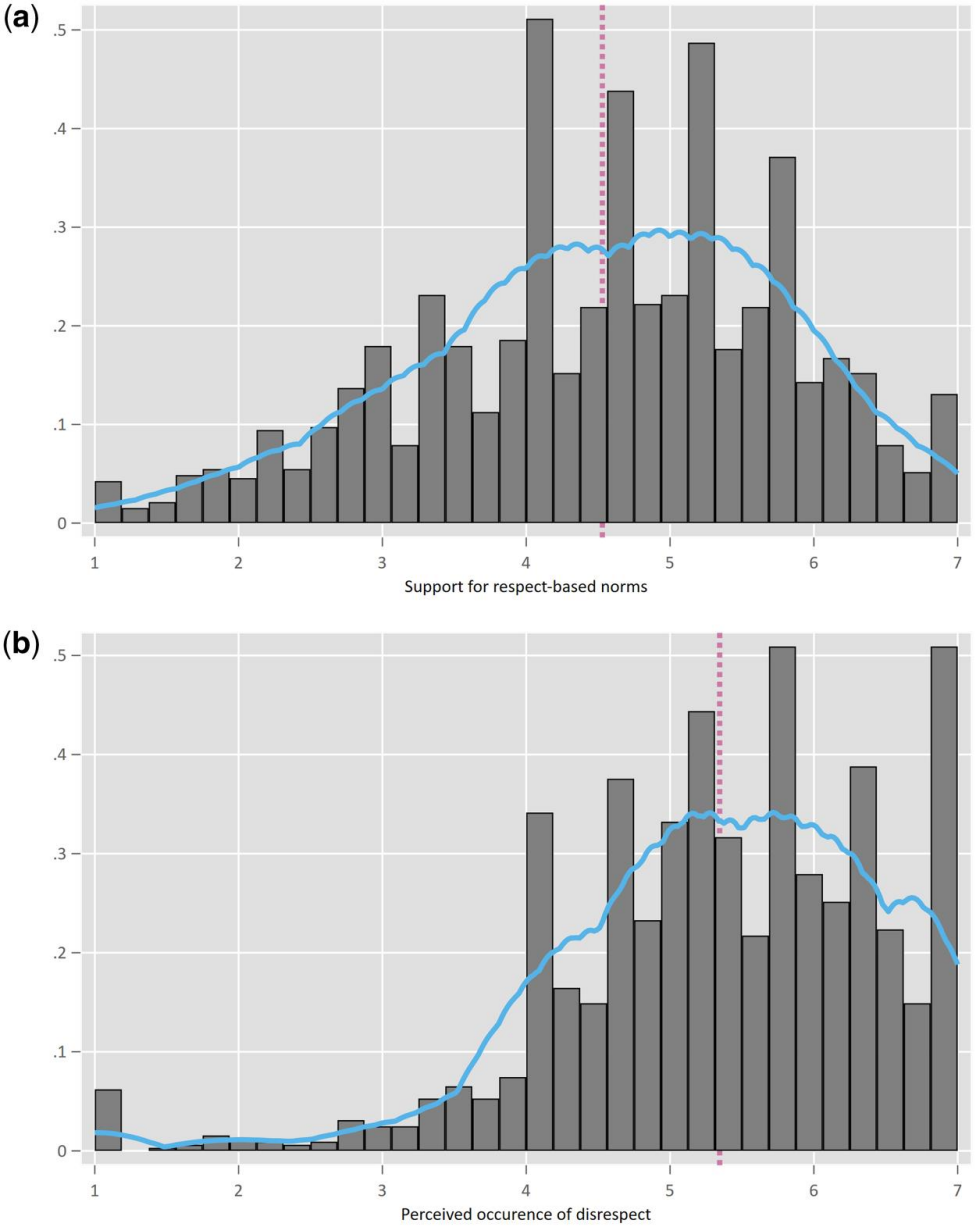
(a) Support for respect-based norms. Distribution of composite norm support variable; kernel density plotted; vertical dotted line represents the mean. Mean 4.529; SD 1.281; Median 4.571; Skew -0.359. (b) Perceived occurrence of disrespect. Distribution of composite perceived occurrence of disrespect; kernel density plotted; vertical dotted line represents the mean. Mean 5.345; SD 1.143; Median 5.429; Skew -0.912.

Before diving into the hypotheses, figure 1a shows the spread of the composite score for norm support. The apparent variation indicates that not everyone has a similar norm concept. Notably, the histogram shows no pronounced negative skew, which would point to strong overall norm support. While the center of the graph is situated slightly on the right side of the scale midpoint of 4, the absence of a peak on the high end of the scale, and a substantial amount of variation across scores on both ends, tells us that not everyone adheres to the same normative ideals for mediated political communication. From figure 1b, we can furthermore see that the distribution for the perceived occurrence of disrespect is more left skewed, meaning that a sizable portion of respondents indicates they perceive disrespect occurring. Hence, while many citizens witness disrespect, not everyone holds the same high level of support for respect-based norms.

### Main Analyses

**Figure 2.**
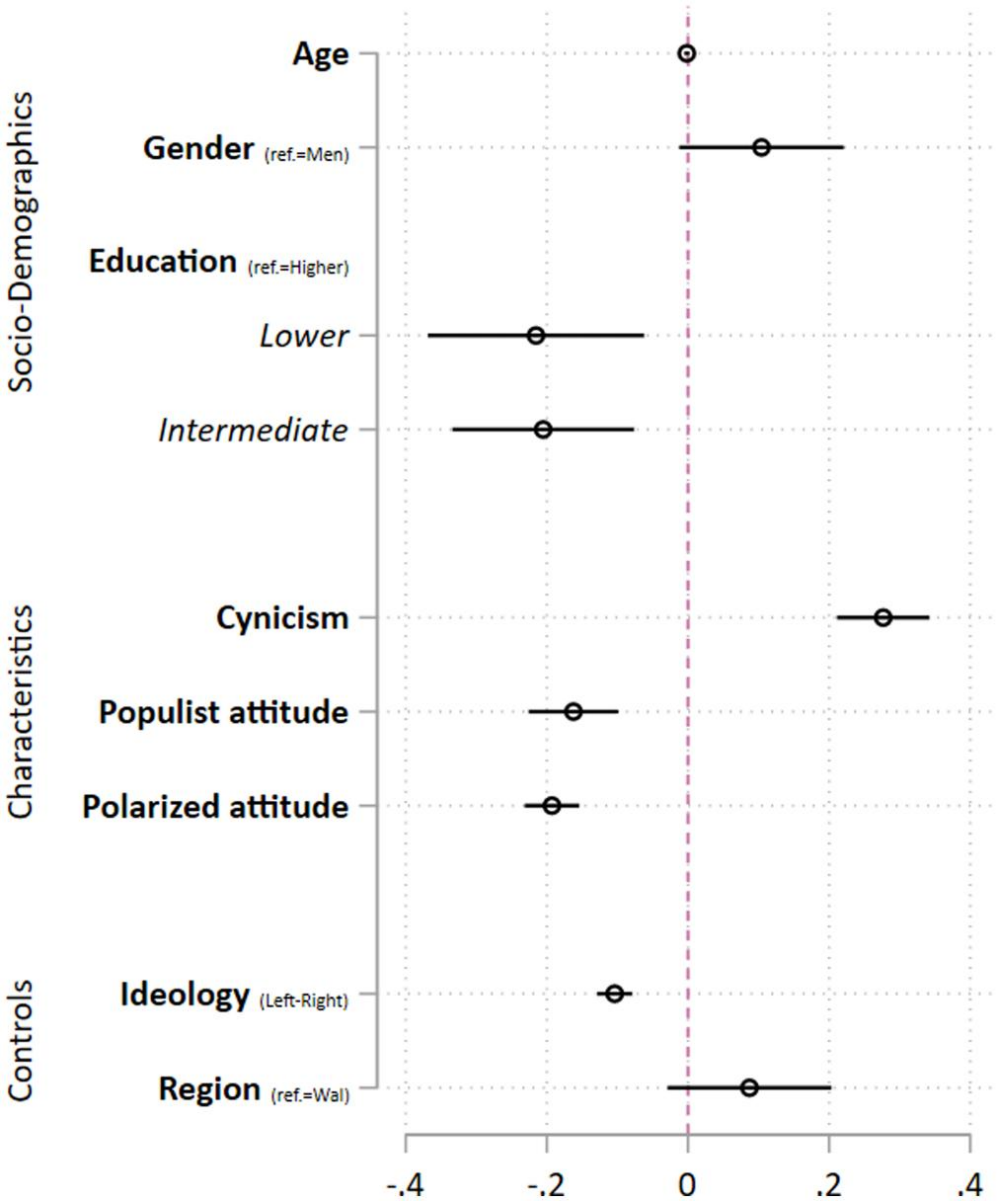
Plotted coefficients for personal characteristics and norm support.

To explore drivers of this variation in norm support, we assess the role of sociodemographics (RQ1) and political attitudes (H1a–c). Regarding RQ1, table 3 and figure 2 show no significant relationship between age and norm support. For gender, we find a marginally significant difference, with women showing stronger norm support (M = 4.619; SD = 1.281) compared to men (M = 4.424; SD = 0.271). Those who attended lower (M = 4.398; SD = 1.325) and intermediate education (M = 4.460; SD = 1.283) support respect-based norms less strongly compared to the higher educated (M = 4.642; SD = 1.251).

**Table 3.** Personal characteristics and norm support.

	Norm support		
	B	se	*p*
Age	−0.002	0.002	0.402
Gender (ref.=Men)			
Women	0.104	0.059	0.079
Education (ref.= Higher)			
Lower	−0.215	0.078	0.006
Intermediate	−0.205	0.066	0.002
Cynicism	0.277	0.033	0.000
Populist attitude	−0.162	0.032	0.000
Polarized attitude	−0.193	0.020	0.000
Ideology	−0.104	0.013	0.000
Region (ref.=Wallonia)	0.087	0.059	0.141
Intercept	5.234	0.177	0.000
R2	0.132		
Adj. R2	0.128		
N	1,739		

*Note*: Estimates are the result of an OLS linear regression; entries are unstandardized coefficients; standard errors; and *p*-values.; For a correlation matrix of predictors, see Supplementary Material section B.

Turning to political attitudes, more cynical citizens report stronger norm support: a one-unit increase in cynicism (1–7 scale) relates to approximately one-fifth of a standard deviation increase in norm support. Conversely, citizens with stronger populist and polarized attitudes show lower norm support, with a one-unit increase in these attitudes corresponding to 0.162 and 0.193 decreases in norm support, respectively. These findings align with H1b (populist attitudes) and H1c (polarized attitudes), but not with H1a (cynicism). Interestingly, norm support also significantly relates to the control variable ideology, with left-leaning respondents exhibiting stronger norm support (see also Conway and Stryker 2021).

**Figure 3.**
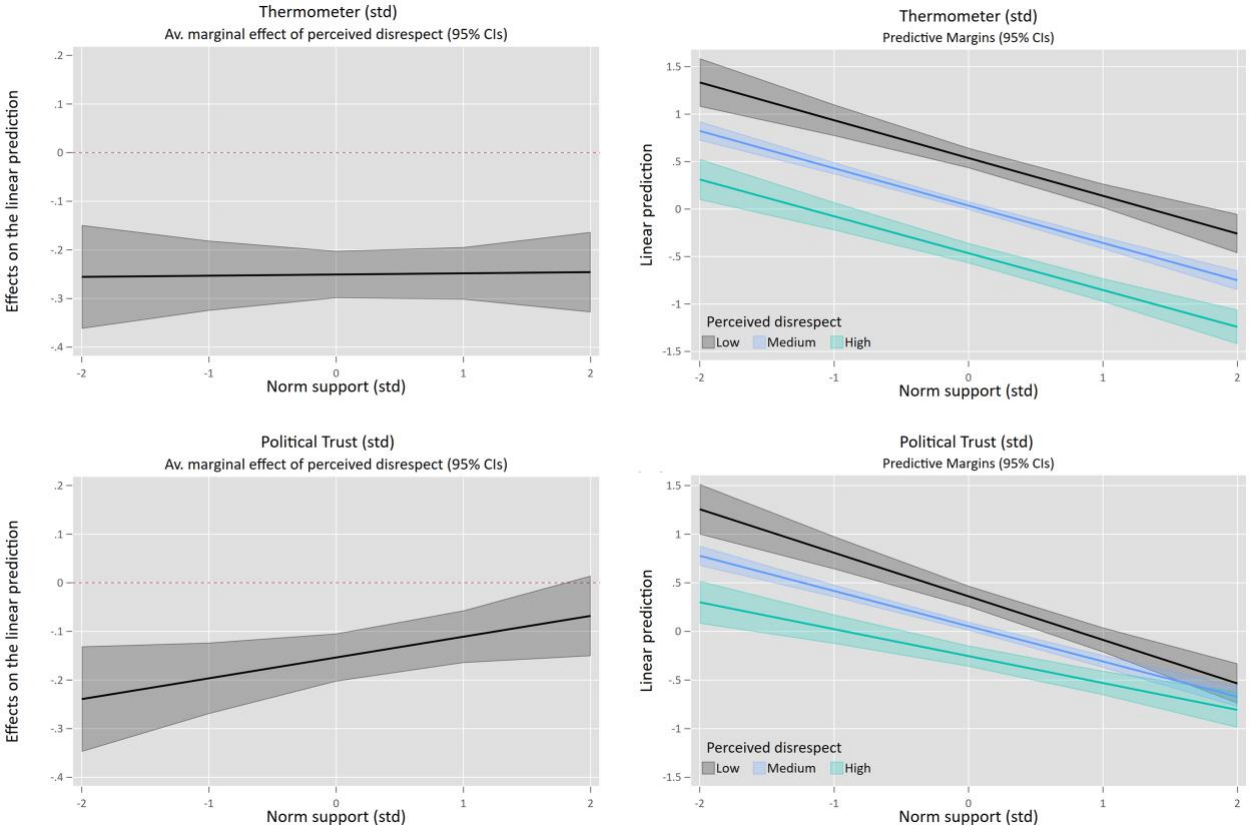
Interaction between norm support and perceived disrespect for feeling thermometer ratings (top row) and political trust (bottom row). Plotted average marginal effects (left) and linear predictions (right) based on OLS regressions (see table 4), using variables standardized around a mean of zero with a standard deviation of 1. The lines in the linear predictions (right hand) correspond to low (2 SD below the mean), medium (mean), and high (2SD above the mean) levels of perceived disrespect.

**Figure 4.**
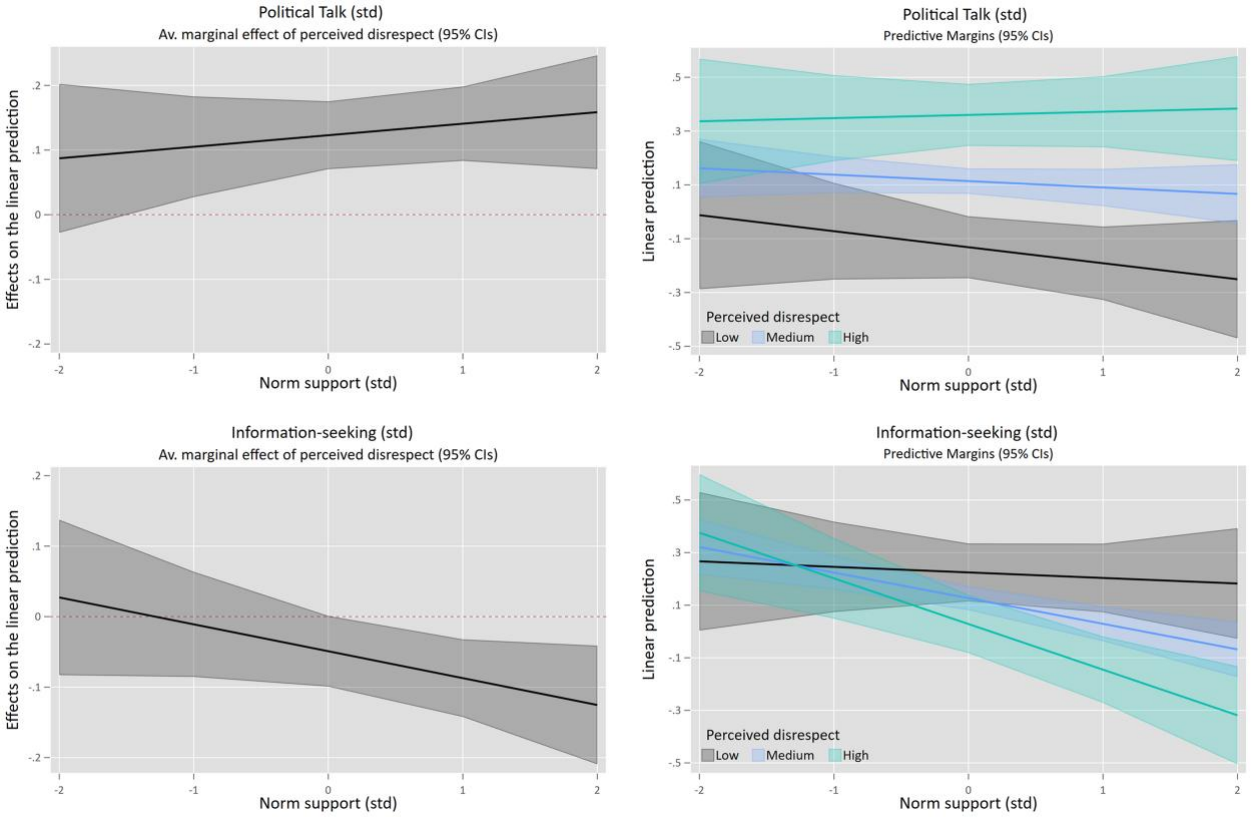
Interaction between norm support and perceived disrespect for political talk (top row) and political information seeking (bottom row). Plotted average marginal effects (left) and linear predictions (right) based on OLS regressions (see table 5), using variables standardized around a mean of zero with a standard deviation of 1. The lines in the linear predictions (right hand) correspond to low (2 SD below the mean), medium (mean), and high (2 SD above the mean) levels of perceived disrespect.

Next, we assess whether this variation in norm support matters, by studying if the relationships between perceived disrespect on the one hand, and the four political outcomes on the other, are moderated by citizens’ norm support (H2a–d). Table 4 shows results for affect toward politicians and political trust; table 5 for political talk and information seeking. Since not all variables use the same scales, all were standardized around a mean of 0 with a standard deviation of 1. To ease interpretation of the interactions, both average marginal effects (AMEs) and linear predictions are plotted in figures 3 and 4 (corresponding tables in Supplementary Material section D).

**Table 4.** Interaction between norm support and perceived disrespect for (a) affect toward politicians and (b) political trust.

	(a) Feeling thermometer (std)						(b) Political trust (std)					
	(1)			(2)			(3)			(4)		
	β	se	*p*	β	se	*p*	β	se	*p*	β	se	*p*
Perceived disrespect (std)	−0.250	0.024	0.000	−0.251	0.025	0.000	−0.138	0.024	0.000	−0.154	0.025	0.000
Norm support (std)	−0.393	0.023	0.000	−0.393	0.023	0.000	−0.351	0.023	0.000	−0.362	0.024	0.000
Perc. disrespect x												
Norm support				0.002	0.021	0.907				0.043	0.021	0.043
Age	0.005	0.001	0.001	0.005	0.001	0.001	0.004	0.001	0.011	0.004	0.001	0.008
Education (ref=int.)												
Lower	−0.127	0.063	0.044	−0.127	0.063	0.045	−0.248	0.064	0.000	−0.242	0.064	0.000
Higher	0.147	0.050	0.003	0.147	0.050	0.003	0.225	0.051	0.000	0.222	0.051	0.000
Gender (ref=men)												
Women	−0.000	0.046	0.998	−0.000	0.046	1.000	−0.010	0.047	0.824	−0.009	0.047	0.842
Ideology (left-right)	−0.018	0.010	0.077	−0.018	0.010	0.078	−0.029	0.010	0.005	−0.028	0.010	0.005
Region (ref=Wallonia)												
Flanders	0.030	0.045	0.511	0.029	0.045	0.513	0.121	0.046	0.008	0.118	0.046	0.010
Intercept	−0.144	0.108	0.181	−0.145	0.108	0.179	0.033	0.110	0.763	0.021	0.110	0.846
R2	0.205			0.205			0.162			0.165		
Adj. R2	0.201			0.200			0.158			0.160		
N	1,582			1,582			1,589			1,589		

*Note:* Estimates are the result of OLS linear regressions using standardized dependent and independent variables.

**Table 5.** Interaction between norm support and perceived disrespect for (c) political talk and (d) information seeking.

	(c) Political talk (std)						(d) Information seeking (std)					
	(1)			(2)			(3)			(4)		
	β	se	*p*	β	se	*p*	β	se	*p*	β	se	*p*
Perceived disrespect (std)	0.129	0.025	0.000	0.123	0.027	0.000	−0.064	0.025	0.010	−0.050	0.026	0.055
Norm support (std)	−0.019	0.025	0.432	−0.024	0.025	0.344	−0.109	0.024	0.000	−0.099	0.025	0.000
Perc. disrespect x												
Norm support				0.018	0.022	0.426				-0.039	0.022	0.076
Age	−0.002	0.002	0.155	−0.002	0.002	0.170	0.009	0.002	0.000	0.009	0.002	0.000
Education (ref=int.)												
Lower	−0.086	0.068	0.206	−0.084	0.068	0.219	−0.102	0.067	0.126	−0.107	0.067	0.108
Higher	0.153	0.054	0.005	0.152	0.055	0.005	0.175	0.053	0.001	0.177	0.053	0.001
Gender (ref=men)												
Women	−0.273	0.050	0.000	−0.272	0.050	0.000	−0.358	0.048	0.000	−0.359	0.048	0.000
Ideology (left-right)	0.022	0.011	0.040	0.022	0.011	0.038	−0.011	0.010	0.314	−0.011	0.010	0.292
Region (ref=Wallonia)												
Flanders	0.201	0.049	0.000	0.200	0.049	0.000	0.016	0.048	0.729	0.019	0.047	0.692
Intercept	0.287	0.117	0.014	0.282	0.117	0.016	−0.185	0.114	0.265	−0.174	0.114	0.309
R2	0.060			0.060			0.085			0.087		
Adj. R2	0.055			0.055			0.080			0.082		
N	1,594			1,594			1,594			1,594		

*Note:* Estimates are the result of OLS linear regressions using standardized dependent and independent variables.

Starting with affect toward politicians, both perceiving more disrespect and more strongly supporting respect-based norms are related to lower thermometer ratings. The two do, however, not seem to interact. Regardless of one’s level of norm support, witnessing a lot of disrespectful communication goes hand-in-hand with more negative feelings toward politicians (AME at -2SD norm support = -0.256; *p *< 0.000, AME at +2SD = -0.246; *p *< 0.000). For political trust, norm support does make a difference. Independently, stronger norm support and higher perceived disrespect relate to lower political trust. Yet counterintuitively, the negative relationship between perceived disrespect and political trust becomes gradually less pronounced as norm support increases, going from an AME of -0.239 at 2SD below the mean of norm support (*p *> 0.000), to -0.111 at 1 SD above the mean (*p *> 0.000) and a nonsignificant relation at 2SD above the mean (AME = -0.068; *p *= 0.106). These findings run counter to the expectations formulated in H2: the relationship between perceived disrespect and affect toward politicians is not contingent on norm support; the relationship between perceived disrespect and political trust is, but not in the way we expected.

Next, we examined if norm support moderates the relationship between perceived disrespect and talking about politics. Although we do not find a significant interaction, the AMEs and visual inspection of the plots (figure 4) seem to suggest that perceiving more disrespect is related to talking about politics with others *more* frequently, except for those with very low (-2SD) levels of norm support (AME = 0.087; *p *= 0.137). For respondents at -1SD to +2SD levels of norm support, there is a positive relation between perceived disrespect and political talk, which gets more pronounced as norm support increases (AMEs ranging 0.087 -0.141; *p *< 0.01).

Finally, we find a marginally significant interaction for political information seeking. Perceiving more disrespect is related to seeking out less information about politics, a relationship that is particularly pronounced for those who support respect-based norms more strongly. In particular, the negative relationship between perceived disrespect and information seeking hits significance at mean levels of norm support (AME = -0.05; *p* = 0.055) and increases as norm support increases (AME at +1SD = -0.089; *p* = 0.002, AME at +2SD = -0.127; *p *= 0.004). Besides showing this negative relation for people who support respect-based norms, the results also indicate that disrespect does not go together with *more* information-seeking behavior for those displaying less norm support. Overall, these findings are partly in line with H2c/d: the relationship between perceived disrespect and discussing politics with others is not moderated by levels of norm support as we expected, but the relation with information-seeking avoidance is, to some extent.

### Additional Analyses

At first sight, our main findings show little support for H2. Rather than showing the expected interactions, Tables 4 and 5 mainly indicate important unmoderated relationships between perceived disrespect, norm support, and political outcomes. First, citizens who perceive lots of disrespect report significantly less affect toward politicians, lower political trust, less information-seeking, and more political talk. Second, citizens with higher norm support show significantly colder affect, lower trust, information seeking, and less talking about politics. In other words: for all measures, direct relations occur for both perceived disrespect *and* norm support, and only for information-seeking does a (marginally) significant interaction effect appear in line with expectations. Yet, before jumping to conclusions, we want to note that we analyzed seven norm manifestations together. Importantly, because some norm manifestations may behave differently than others, some meaning may have been lost and certain associations dampened or changed. While it is beyond the scope and space constraints of this paper to extensively dive into each separate norm, Supplementary Material section F shows analyses for all separate norms, pointing to some noteworthy differences from the main analyses.

In several cases, the relationship between perceived disrespect and the dependent variables *is* significantly moderated by norm support, thereby nuancing some results from the main analyses and indicating that the H2 expectations hold for several separate norm manifestations (but not for others). While our data, as well as previous studies, show the existence of an overarching construct of respect-based norms, making it a valid decision to scale them, these additional analyses do indicate that it remains important to also study different respect-based norms separately, to get more detailed insights. For instance, the main analysis showed no significant interaction for affect toward politicians, and even a positive interaction for political trust. However, norm support does significantly moderate the relation between perceived disrespect and these outcomes in the expected (negative) direction for several separate norms, such as truthfulness and politeness.

Examining then the individual norms for RQ1 and H1a–c (variation in norm support), we see, overall, a more consistent pattern in line with the findings above, especially for the role of education and cynical, populist, and polarized attitudes. For gender and age, results vary somewhat more across norms: the politeness and understandability norm in particular are supported more strongly by women; and support for politeness, engaging with other standpoints, and justifying arguments is higher for younger people. Older people more strongly support truthful, to-the-point, and understandable communication.

Finally, we also reran the main analyses with additional control variables, including political interest and frequency of exposure to political discussions (Supplementary Material section E). Overall, adding these controls revealed similar patterns to those elaborated upon in the main analyses, except for three findings: the marginally significant relation between gender and norm support disappears (RQ1), the unexpected positive interaction effect on political trust turns insignificant (H2a), and the marginally significant negative interaction effect on political information seeking becomes stronger (*p *= 0.025; supporting H2d).

## Discussion

Worries about the state of (mediated) political communication are oftentimes grounded in the assumption that ideals for political communication, such as well-reasoned, civil, and understandable communication (*respect-based norms*), are violated frequently by politicians, yet shared widely among the public. However, empirical insight into public support for respect-based norms is surprisingly scarce. Do people indeed widely share support for these norms, or is there variation across the public? And does variation in norm support moderate the relationships between norm violations and democratic outcomes, like political trust or political information seeking? Focusing on the context of mediated political discussions, this study aimed to address these two main questions.

As expected, not everyone adheres to the same political communication standards. While many people regularly perceive deviations from respect-based norms, this is not equally problematic to everyone and, as such, may not always have equal repercussions. Regarding RQ1, we find evidence of stronger norm support among higher-educated people, but no age-based differences. For gender, results suggest a small difference, with women supporting respect-based norms more than men, yet additional analyses showed that this overall finding in the composite measure was driven predominantly by support for the politeness norm. Some of these findings are in line with previous work, reporting for instance stronger adherence to deliberative norms among higher-educated people, but no relation with age (Jennstål, Uba, and Öberg 2021). Yet other findings are not corroborated by previous research, which reports women to be generally less adherent to deliberative discussion norms (Jennstål, Uba, and Öberg 2021) and finds older people to be more accepting of disrespect in politics (Mölders and Van Quaquebeke 2017). These differing results seem to point to the importance of contextual factors, such as communicative venue, actor (e.g., politicians versus citizens), and country, as well as the specific norm(s) one studies.

In line with expectations, our results furthermore show that those with stronger populist and polarized attitudes are less supportive of respect-based norms (H1b, c). This lower level of norm support may explain why populist citizens seem to be more accepting of the—generally more simplified and impolite—communication style of populist politicians (Moffitt 2016), and also connects to existing work suggesting that citizens with stronger populist and polarized attitudes show less support for *democratic* norms (Oswald and Robertson 2020; Plescia and Eberl 2021). Populist and polarized citizens may see violation of communication norms as a means, which is justified by the end of their side defeating the “out-group.” Hence, contrary to scholarly expectations of how politicians should behave,^6^ at least part of the citizenry may perceive disrespect as (an essential) “part of the political game.”

Additionally, and in contrast to our expectations (H1a), cynical citizens adhere more strongly to respect-based norms. It might be that these citizens have high normative standards to which they hold politicians, but low expectations for these norms to be met: when your standards are higher, it is easier to be disappointed. This way, strong norm support may even precede cynicism: when frequently exposed to norm violations, especially those attaching great importance to these norms may become more politically cynical. The causal mechanisms underlying this finding need further examination in future research, to establish whether, for instance, exposure to disrespect heightens cynicism for citizens with higher levels of norm support.

As critics of norm-violating political communication tend to point to its detrimental democratic consequences, it is important to assess whether those consequences could be different for citizens with stronger or weaker norm support. First, building on cross-sectional data and hence being careful with causal claims, our results show *direct* negative relationships between norm support and all studied outcomes. The stronger one supports respect-based norms, the lower they score on affect toward politicians, political trust, information seeking, and political talk. This indicates that, even independently from perceived violations, norm support matters for these outcomes. Norm support may thus be a relevant variable to incorporate in future work studying disrespect. Additionally, frequently perceiving norm-violating communication is negatively related to citizens’ affect toward politicians, political trust, and political information seeking, and positively related to talking about politics with others (again, *direct* relationships). This is in line with existing worries about the negative consequences of disrespect, and with existing work showing that norm-violating communication negatively influences outcomes like political trust and candidate evaluations but can also capture the audience’s attention (e.g., Mutz 2015). Do differences in norm support then matter for these relationships? Overall, our answer to this question is a nuanced and tentative yes.

First, the main analysis found no significant interaction between norm support and perceived disrespect for affect toward politicians. Regularly perceiving disrespect and strongly supporting respect-based norms both relate to colder feelings toward politicians, independently of one another. Second, the relationship between perceived disrespect and political trust was found to be contingent upon norm support yet opposing the expected direction; for those with very high levels of norm support, the actual occurrence of disrespect mattered less for their political trust. One potential explanation can be sought in the overall lower levels of political trust among strong norm supporters. These people have high standards for political communication and may therefore be more sensitive to violations. Even infrequent violations could thus have a more substantial impact on their political trust levels, causing it to be low across different levels of perceived disrespect. Another explanation may relate to the grouping together of the separate norms. The additional analyses (Supplementary Material section F) showed that the individual norms may still bear substantial meaning on their own, which may get lost by scaling them. Indeed, looking at the different norms separately, the expected interactions for norm support do appear for some individual norms, and we see this for both political trust and affect toward politicians.

Third, no significant interaction effect between norm support and perceived disrespect was found for talking about politics. While the average marginal effects did hint at some differences at very low levels of support, the results overall show that witnessing more norm-deviating communication relates to *more* frequent political talk. This aligns with Chen’s (2017)  *defense effect* and with the frequently discussed attention-grabbing potential of norm violations: when someone breaks the “rules,” that is something to talk about (Mutz 2015). Finally, in line with expectations, we did find more norm-adhering citizens to be less likely to seek out political information when perceiving more disrespect, implying a tuning out of politics. Interestingly, results also indicated that citizens with *lower* levels of norm support do not seek out political information *more* when perceiving more disrespect. Breaking the norms is thus not only harmful for the political engagement of norm-adhering citizens; it does not seem to positively impact nonadhering citizens to seek out more information either.

Based on all this, we cautiously conclude from this study that norm support could be *one* of the important factors that may explain conditional effects previously found in the literature, showing that effects of politicians’ use of norm-violating communication vary across citizens with different personality traits and political attitudes (e.g., Mutz and Reeves 2005; Nai and Maier 2021; Vargiu and Nai 2022). However, nuance is crucial here, as the importance of norm support in these relationships seems to depend substantially on the outcome and specific norms we study. Overall, this study showed that support for the overarching construct of respect-based norms matters for the relationship between perceived disrespect and political information seeking but less so for affect toward politicians, political trust, and political talk. Yet, this changes when we look at the individual norms, showing for instance that citizens’ level of support for specific norms of politeness, truthfulness, and engagement with other standpoints does influence these outcomes.

This brings us to the first limitation of this study. Drawing on previous literature and exploratory factor analysis confirming the existence of an overarching construct of respect-based norms, we presented a first step to map variation in citizens’ norm support. On the one hand, this allows for a first overall picture of respect in political communication as a nonuniversal norm, and including different shades of respect-based norms makes for an encompassing impression. On the other: by grouping separate norm manifestations, the main analyses disregard potential nuances. While providing first insights into the individual norms, it is important for future work to dig deeper into different respect-based norms in a more fine-grained manner. If we want to understand exactly under what circumstances which types of disrespect have harmful democratic effects, more research is needed.

Second, using cross-sectional survey data, we cannot draw strong conclusions about causality. While we believe our theoretical argument is sensible and substantially grounded in previous literature, we cannot rule out that relationships may also work in opposite directions; for instance, lower levels of political trust or higher support for respect-based norms leading to more perceived instances of disrespect. To further unpack the (causal) relationships between perceived norm violations, norm support, and democratic outcomes, more (experimental/longitudinal) studies are needed, building on work that already examines various factors influencing perceptions and effects of norm violations (e.g., Muddiman 2017; Kim and Gonzales 2022). Such follow-up work would ideally manipulate politicians’ use of norm violations as well as citizens’ norm support levels (e.g., through priming tasks emphasizing or de-emphasizing the importance of respect-based communication).

Third, mapping and discussing precise *levels* of support for (different) respect-based norms was not the aim of this study. Rather, we intended to showcase the presence and importance of individual variation in norm support, which we believe to be relevant across contexts. Yet, as social norms are culturally dependent (Otto, Lecheler, and Schuck 2020; Eriksson et al. 2021), we remain careful in generalizing the exact patterns of norm support displayed here to other contexts. We recommend future work to employ larger-scale cross-national designs to map differences and similarities in the presence and effects of norm support. Additional research is, moreover, needed to better understand norm support in relation to individual-level characteristics, communicators, and settings, other than the ones we studied, thereby examining, for instance, the impact of personality traits, issue under discussion, or debate venue (see also Muddiman, Flores, and Boyce 2022).

Limitations notwithstanding, the findings presented in this study underscore the importance of acknowledging individual-level variations in support for respect-based norms in political communication. This is an initial step toward a better understanding of different responses to, and mixed effects of, disrespect in politicians’ communication. By expanding our gaze beyond a narrow understanding of disrespect as impoliteness alone, we hope to present a richer image of the different ways in which politicians can bend or break the rules, how citizens feel about this, and why that matters.

## Supplementary Material

nfaf001_Supplementary_Data

## Data Availability

Replication data and documentation are available at https://osf.io/5qky2/.

## Footnotes

1 While it is equally important to study disrespectful communication toward the democratic polity, we limit this study’s scope to disrespectful communication toward one’s interlocutor and audience, focusing more on the interpersonal dimension of disrespect.

2 Note that different normative views on the desirability of respectful communication in politics exist in democratic theory, too (cf. Mouffe 1999; Young 2000; Dryzek 2002).

3 Incivility scholars (e.g., Papacharissi 2004; Muddiman 2017; Rossini 2022) often differentiate between interpersonal impoliteness (*personal-level incivility*) and incivility that harms democratic principles (*public-level incivility* or *intolerance*). As indicated in footnote 1, we focus primarily on the former. We are aware of likely overlap between both concepts, which warrants further investigation but falls beyond this study’s scope.

4 Positive effects of incivility have also been reported, for example, increased entertainment and political engagement (Mutz and Reeves 2005; Brooks and Geer 2007).

5 Information about the project and involved universities: https://represent-project.be/.

6 Yet there are also voices in the literature arguing more generally that we should not get rid of incivility in politics (e.g., Chen et al. 2019).
