# Supplementary material for: Different Standards: Observing Variation in Citizens’ Respect-Based Norms for Mediated Political Communication
Source: Public Opin Q. 2025 May 20;89(1):155–85. doi: 10.1093/poq/nfaf001 (PMC12166978; doi:10.1093/poq/nfaf001)

Supplementary Material

Different standards: Observing variation in citizens' respect-based norms for mediated political communication

Emma Turkenburg[1] (*Wageningen University & Research*), Ine Goovaerts (*University of Antwerp*), Sofie Marien (*KU Leuven*)

**A**	**Survey information**
A1	Survey ordering
A2	Survey wording
**B**	**Descriptives**
B1	Sample descriptives
B2	Descriptives for core variables
B3	Distribution of dependent variables
B4	Correlation matrix of core variables
B5	Correlation between norm-support items and perceived disrespect items
**C**	**Exploratory factor analyses**
C1	Exploratory factor analysis norm-support: nine separate norm-manifestations
C2	Exploratory factor analysis norm-support: seven separate norm-manifestations
C3	Exploratory factor analysis perceived disrespect: nine items
**D**	**Average Marginal Effects**
D1	The relation between perceived disrespect and thermometer ratings
D2	The relation between perceived disrespect and political trust
D3	The relation between perceived disrespect and political talk
D4	The relation between perceived disrespect and information-seeking
**E**	**Analyses with additional control variables**
E1	Variation in norm-support – Extra controls
E2	Interactions – Extra controls
E3	Linear predictions and marginal effects – Extra controls
**F**	**Analyses for separate norm manifestations**
F1	Variation in norm-support – Separate norms – Plotted coefficients
F2	Variation in norm-support – Separate norms
F3	Interactions – Separate norms
F3	Linear predictions and marginal effects – Separate norms

[1] Corresponding author: emma.turkenburg@wur.nl

A. Survey information

A1. Survey ordering

Part of larger survey with blocks of questions related to other research / relevant blocks & relevant questions / bold variables used in some capacity

Survey block	Questions from block used (in order of appearance in survey)
Block 1. General	Introduction and informed consent<br>Region | Age | Gender | Education | Political interest | Ideology | Satisfaction with democracy | Political trust
Block 2. Participatory processes	*No questions from this block were used*
Block 3. Political discussion and debate	Frequency of exposure to political discussions | Norm-support | Perceived violations | Thermometer politicians generally | Information-seeking battery | Political talk | Cynicism | Populist attitudes | Affectively polarized attitudes
Block 4. Solutions	*No questions from this block were used*
Block 5. Climate policy	*No questions from this block were used*
Block 6. VAAs	*No questions from this block were used*
Block 7. Covid	*No questions from this block were used*

A2. Survey wording (translated from Dutch/French questionnaire)

Variable	Question	Type	Answers
Region	What is your main region of residence?	Multiple choice	Flanders region | Wallonia region | Brussels region *[directed to end of survey]*
Age	What is your age?	Drop-down	
Gender	What is your gender?	Multiple choice	Man | Woman | Non-binary | Prefer not to say
Education	What is your highest level of education?	Multiple choice	No or primary education | Secondary education, not fully completed (lower ASO, BSO or TSO) | Secondary education, fully completed (ASO, BSO or TSO) | Higher non-university education | University education
Political interest	To what extent are you interested in politics in general?	0-10 scale	Not interested at all (0) – Very interested (10)
Ideology	The terms 'left' and 'right' are often used in politics. Can you place your own views on a scale of 0 to 10, where 0 means 'left', 5 means 'in the center' and 10 means 'right'?	0-10 scale	Left (0) – In the center (5) – Right (10)
Satisfaction with democracy	In general, how satisfied are you with the way democracy works in Belgium?	0-10 scale	Extremely dissatisfied (0) – Extremely satisfied (10)

Political Trust	Please indicate on a scale of 0 to 10 how much you personally trust each of the following institutions?<br>(1) Political parties<br>(2) Federal parliament<br>(3) Politicians<br>(4) Federal government		No trust at all (0) – Trust completely (10)
Introduction to the block with questions on political discussion and debate	Politicians regularly appear in the **media to discuss politics with each other**, for instance in talk shows, news programs and election debates. In what follows, we will ask your **opinion and expectations of the communication of politicians in such media discussions**. There are no right or wrong answers, we are simply interested in your opinion.		
Frequency of exposure to political discussions	How often do you watch or listen to such political discussions?	Multiple choice	Never | Less than once a month | Once a month | Several times a month | Once a week | Several times a week | Daily
Norm-support	In what follows, we aim to gauge your opinion on **how you would like politicians to communicate in political discussions in the media**. Please indicate, using a 7-point scale, to what extent you agree with the statements below.<br>**In a political discussion**...<br>(1) Politicians do not always have to tell the truth, they are allowed to speculate.<br>(2) Politicians do not always have to be respectful, a firm lashing out is allowed.<br>(3) Politicians do not always have to let others finish when speaking; interrupting each other is part of the job.<br>(4) Politicians do not always have to respond to the views of others, they should mainly communicate their own ideas.<br>(5) Politicians do not always have to be "to the point" and concrete, it is okay if they digress.<br>(6) Politicians should be careful with difficult language, they should always communicate in a comprehensible way that is understandable to everyone.<br>(7) Politicians should not simplify matters, they can go deeper into more difficult topics.	1-7 scale	Completely disagree (1) – Completely agree (7)<br>I don’t know

	(8) Politicians should primarily communicate what they stand for, justification and argumentation for their positions are not always necessary.<br>(9) Politicians do not have to discuss everything at length, one-liners and slogans are part of the game.		
Perceived disrespect	We would now like to ask you to indicate to what extent these statements are applicable to what you think political discussions in the media look like **today**.<br>**Today, I see that in political discussions**...<br>(1) Politicians do not tell the truth, there is frequent speculation.<br>(2) Politicians are not respectful, they often lash out at each other.<br>(3) Politicians do not let others finish speaking and they often interrupt each other.<br>(4) Politicians do not address the views of others and they mainly communicate their own ideas.<br>(5) Politicians are not "to the point" and concrete, but often digress.<br>(6) Politicians do not communicate in a way that is understandable for everyone.<br>(7) Politicians simplify things and do not go deep into more difficult topics.<br>(8) Politicians do not give justification and argumentation for their positions but only say what they stand for.<br>(9) Politicians often use one-liners and slogans.	1-7 scale	Completely disagree (1) – Completely agree (7)<br>I don't know
Affect towards politicians	We would now like to ask you to indicate your feelings about politicians in general on a thermometer scale of 0-100 degrees. A score of "0" represents cold feelings, "50" represents neutral, and "100" represents warm feelings.	0-100 slider	Cold (0) – Neutral (50) – Warm (100)
Information-seeking	We would now like to ask you to indicate to what extent you agree with the following statements.<br>1. I closely follow what is going on in politics.<br>2. Political programs in the media do not interest me. [rev]<br>3. When I see politicians debating, I tune out. [rev]<br>4. I deliberately search for political news programs.	1-7 scale	Strongly Disagree (1) –Strongly Agree (7)

Political talk	Sometimes people talk about politics with others (such as friends, family, colleagues). How often do you discuss politics with other people?	Multiple choice	Never | Less than once a month | Once a month | Several times a month | Once a week | Several times a week | Daily
Cynicism	To what extent do you agree with the statements below?<br>1. Politicians deliberately promise more than they can deliver.<br>2. Politicians mainly act out of self-interest.<br>3. Politicians do not understand what matters to the people.	1-7 scale	Strongly Disagree (1) –Strongly Agree (7)
Populist attitudes	To what extent do you agree with the statements below?<br>1. Politicians in parliament must follow the will of the people.<br>2. Politicians talk too much and act too little.<br>3. The most important political decisions should be made by the people and not by politicians.<br>4. I would rather be represented by an ordinary citizen than by a professional politician.	1-7 scale	Strongly Disagree (1) –Strongly Agree (7)
Affectively polarized attitudes	To what extent do you agree with the statements below?<br>1. There are people I have come to dislike for their views.<br>2. You can tell if someone is good or bad based on their political affiliations.<br>3. I tend to avoid some people because of their opinions.	1-7 scale	Strongly Disagree (1) –Strongly Agree (7)

B. Descriptives

B1. Sample descriptives

	Sample		Belgian population
	%	N	%
Region			
Flemish	50	1015	57.92%
Walloon	50	1015	31.47%
Total	100	2030	89.39%
Gender			
Male	47.64	967	49.25 %
Female	51.87	1053	50.75 %
Non-binary	0.39	8	No data
Prefer not to say	0.10	2	No data
Total	100	2030	
Age			% of adult pop.
18-34	25.76	523	26.14%
35-54	34.53	701	32.56%
55-74	36.95	750	29.65%
75+	2.76	56	11.65%
Total	100	2030	100
Mean (SD)	47.88 (16.68)		
Education level			
Lower	22.02		21.71%
No or primary school	4.68	95	
Secondary, unfinished	17.34	352	
Intermediate			38.04%
Secondary, finished	35.02	711	
Higher	42.95		40.24%
Higher, non-university	27.68	562	
Higher, university	15.27	310	

Source Belgian population data: Statbel, population data for 2023, education data for 2022

B2. Descriptives for core variables

Variable	Mean	SD	Min	Max	N	Cronbach's α
Norm-support composite (7 norms)	4.529	1.281	1	7	1752	0.802
Truth	5.413	1.868	1	7	1902	
Polite	4.222	1.939	1	7	1889	
No interruption	4.823	2.050	1	7	1894	
Engagement w/ other standpoints	3.608	1.832	1	7	1872	
To-the-point	5.154	1.826	1	7	1899	
Understandable	5.593	1.567	1	7	1874	
Elaborate/No simplification	5.186	1.581	1	7	1873	
Justification	3.890	1.925	1	7	1873	
No oneliners	4.635	1.887	1	7	1873	
Perceived disrespect composite (7 norms)	5.345	1.143	1	7	1718	0.885
Lies/No truth	5.537	1.438	1	7	1858	
Impolite	5.055	1.540	1	7	1869	
Interruption	5.588	1.433	1	7	1876	
No engagement w/ stp of others	5.172	1.533	1	7	1849	
Not to-the-point	5.504	1.444	1	7	1855	
Not understandable	5.163	1.535	1	7	1868	
Not elaborate/Simplified	5.175	1.504	1	7	1839	
No justification	5.155	1.505	1	7	1840	
Oneliners	5.377	1.445	1	7	1834	
Political cynicism	5.458	1.288	1	7	2029	0.833
Populist attitudes	5.071	1.292	1	7	2028	0.838
Polarized attitudes	3.905	1.526	1	7	2029	0.756
Feeling thermometer	41.394	23.132	0	100	2015	
Political trust	4.040	2.427	0	10	2024	0.959
Talk about politics	2.956	1.596	1	7	2030	
Political Information-seeking	3.679	1.420	1	7	2029	0.770

Note. Variation in N is due to don't know and missing answers.

B3. Distribution of dependent variables

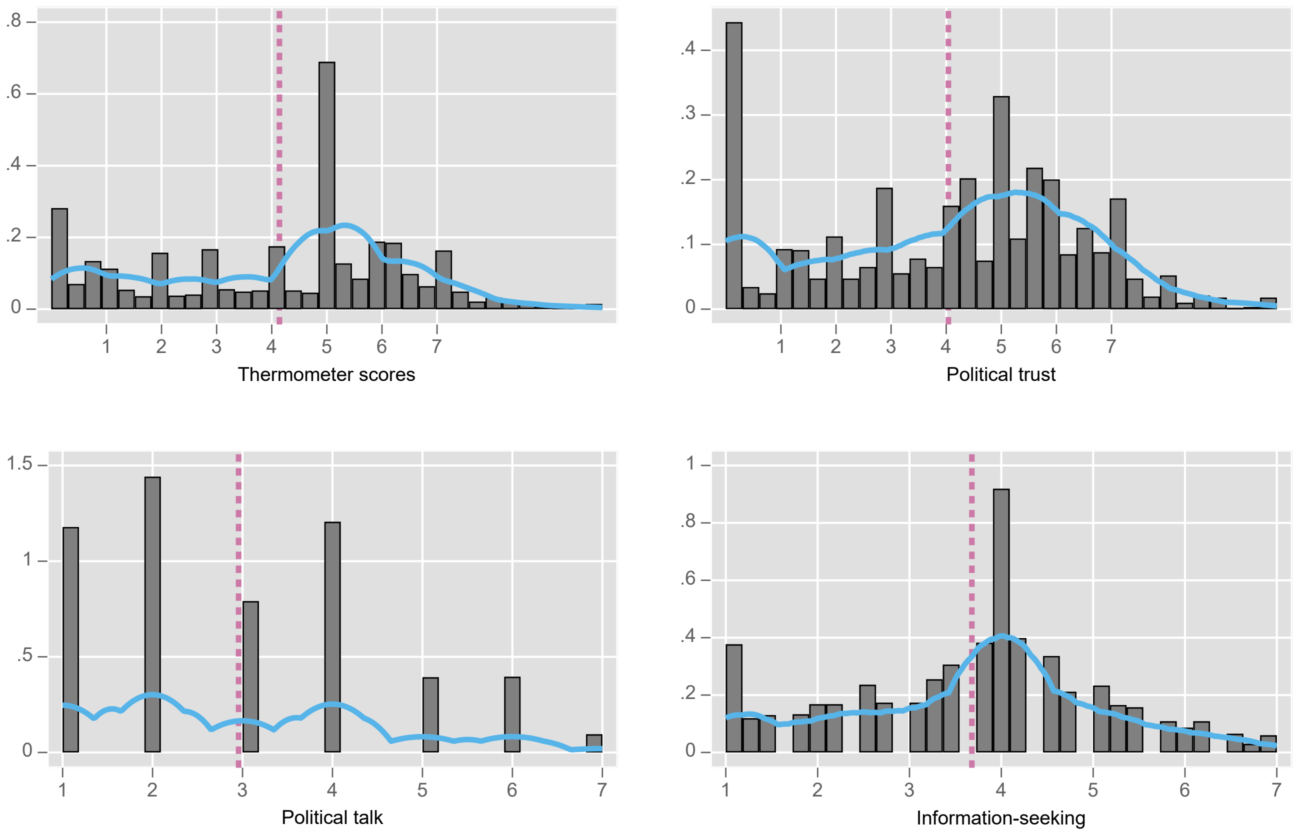

Note. Distributions of dependent variables; kernel density plotted in blue; means in red.

B4. Correlation matrix of core variables

Variables	1	2	3	4	5	6	7	8	9	10	11	12	13
1. Norm-support	1.000												
2. Perc. disrespect	-0.042<br>*0.093*	1.000											
3. Cynicism	0.094<br>*0.000*	0.568<br>*0.000*	1.000										
4. Populist att.	-0.050<br>*0.035*	0.457<br>*0.000*	0.711<br>*0.000*	1.000									
5. Polarized att.	-0.230<br>*0.000*	0.133<br>*0.000*	0.145<br>*0.000*	0.181<br>*0.000*	1.000								
6. Age	0.021<br>*0.372*	0.281<br>*0.000*	0.245<br>*0.000*	0.120<br>*0.000*	-0.043<br>*0.053*	1.000							
7. Education	0.079<br>*0.001*	-0.022<br>*0.370*	-0.081<br>*0.000*	-0.144<br>*0.000*	0.006<br>*0.800*	-0.147<br>*0.000*	1.000						
8. Gender	0.076<br>*0.001*	-0.035<br>*0.145*	-0.015<br>*0.503*	-0.004<br>*0.842*	-0.067<br>*0.003*	-0.201<br>*0.000*	0.025<br>*0.253*	1.000					
9. Thermometer	-0.373<br>*0.000*	-0.219<br>*0.000*	-0.409<br>*0.000*	-0.352<br>*0.000*	0.043<br>*0.052*	-0.010<br>*0.649*	0.072<br>*0.001*	-0.032<br>*0.155*	1.000				
10. Trust	-0.324<br>*0.000*	-0.117<br>*0.000*	-0.362<br>*0.000*	-0.317<br>*0.000*	0.051<br>*0.021*	-0.014<br>*0.542*	0.155<br>*0.000*	-0.028<br>*0.203*	0.694<br>*0.000*	1.000			
11. Pol. Talk	-0.050<br>*0.036*	0.113<br>*0.000*	0.019<br>*0.393*	-0.008<br>*0.734*	0.105<br>*0.000*	0.012<br>*0.600*	0.130<br>*0.000*	-0.157<br>*0.000*	0.147<br>*0.000*	0.144<br>*0.000*	1.000		
12. Info-seeking	-0.119<br>*0.000*	-0.040<br>*0.097*	-0.213<br>*0.000*	-0.229<br>*0.000*	0.045<br>*0.043*	0.137<br>*0.000*	0.104<br>*0.000*	-0.222<br>*0.000*	0.338<br>*0.000*	0.277<br>*0.000*	0.507<br>*0.000*	1.000	
13. Ideology	-0.196<br>*0.000*	0.016<br>*0.515*	0.066<br>*0.003*	0.081<br>*0.000*	0.069<br>*0.002*	0.012<br>*0.590*	-0.005<br>*0.834*	-0.092<br>*0.000*	0.068<br>*0.002*	0.018<br>*0.409*	0.087<br>*0.000*	0.029<br>*0.188*	1.000
14. Region	0.023<br>*0.339*	-0.005<br>*0.820*	-0.016<br>*0.460*	0.032<br>*0.145*	-0.191<br>*0.000*	-0.014<br>*0.527*	-0.003<br>*0.887*	0.003<br>*0.891*	-0.025<br>*0.266*	-0.078<br>*0.000*	-0.088<br>*0.000*	-0.021<br>*0.344*	-0.124<br>*0.000*

Note. Pairwise correlation coefficients and *p-values*

B5. Correlation between norm-support items and perceived disrespect items

	Correlation with corresponding perceived-disrespect item	
**Norm-support item**	*r*	*p*-value
Composite measure	-0.042	*0.093*
Truthful	0.138	*0.000*
Politeness	-0.007	*0.756*
No interruptions	0.083	*0.000*
Engaging with other standpoints	-0.047	*0.045*
To-the-point	0.126	*0.000*
Understandable	0.373	*0.000*
No simplifications	0.289	*0.000*
Justified	-0.097	*0.000*
No one-liners	0.066	*0.005*

C. Exploratory factor analyses

For the factor analyses below, we first report the results of several tests checking whether conducting a factor analyses with these variables is appropriate. Next, we determine how many factors to extract with the factor analysis. We do so in two ways. Firstly, we create a screeplot based on a principle component analysis (PCA), and look at how many components have an Eigenvalue above 1 and are situated "above the elbow". Secondly, we run and plot a parallel analysis with randomly generated eigenvalues, looking at how many factors are higher in the original PCA than in the generated parallel analysis, also indicated in the plot as the factors above the dotted line. After this step, we run the factor analysis, extracting the specified number of factors. When more than one factor is extracted, we rotate the factor loadings matrix. Lastly, we run reliability tests for the items that load well on the extracted factors, to check if they form reliable scales.

C1. Exploratory factor analysis norm-support: nine separate norm-manifestations

Test of appropriateness

Determinant of the correlation matrix	0.117
Bartlett's test of sphericity	*p*=0.000
KMO	0.834

Determining the number of factors to extract

Screeplot after principal component analysis	Plot after parallel analysis
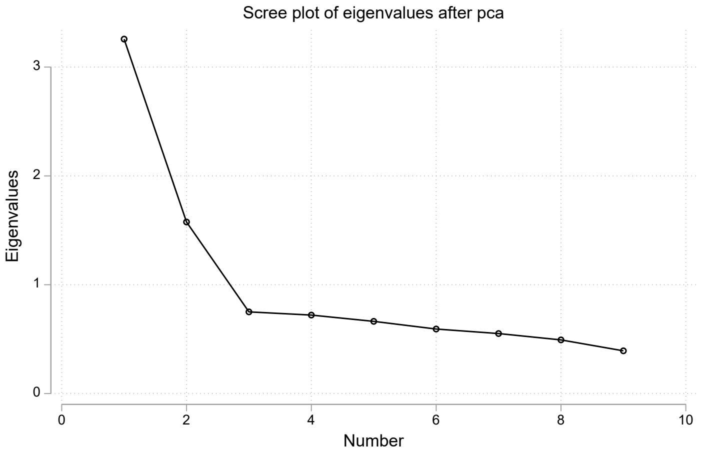	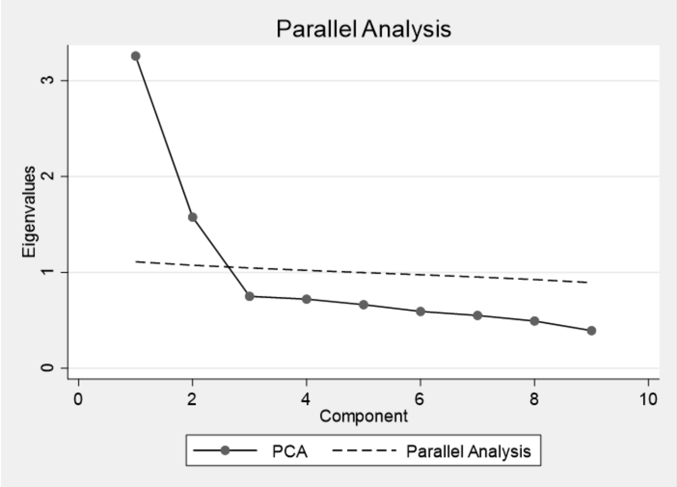
Note. A first PCA results in two factors with eigenvalues above 1.0 (3.257 for factor 1; 1.577 for factor 2)	*Note.* Parallel analysis with randomly generated eigenvalues results in two factors.

Factor analysis (principal factors extraction)

Factor	Eigenvalue	Difference	Proportion	Cumulative
Factor1	2.618	1.818	0.959	0.959
Factor2	0.799	0.788	0.293	1.252
Factor3	0.011	0.016	0.004	1.256
Factor4	-0.004	0.072	-0.002	1.255
Factor5	-0.076	0.043	-0.028	1.227
Factor6	-0.119	0.008	-0.044	1.183
Factor7	-0.127	0.024	-0.047	1.137
Factor8	-0.152	0.069	-0.056	1.081
Factor9	-0.221	.	-0.081	1.000

Rotated factor loadings

Oblique rotation with kaiser normalization, specifying a minimum eigenvalue of 0.7 and retention of two factors

Variable	Factor1	Factor2	Uniqueness
norm_1	0.734	0.138	0.463
norm_2	0.519	-0.184	0.677
norm_3	0.652	0.014	0.576
norm_4	0.334	-0.361	0.734
norm_5	0.741	0.116	0.454
norm_6	0.079	0.575	0.672
norm_7	-0.024	0.492	0.755
norm_8	0.482	-0.244	0.685
norm_9	0.656	-0.021	0.567

Scale reliability

Factor 1 (norm 1, 2, 3, 4, 5, 8, 9)	Cronbach's alpha	0.802
Factor 2 (norm 6, 7)	Spearman-brown coefficient	0.527

C2. Exploratory factor analysis norm-support: seven separate norm-manifestations

Test of appropriateness	
Determinant of the correlation matrix	0.156
Bartlett's test of sphericity	*p*=0.000
KMO	0.861

Determining the number of factors to extract

Screeplot after principal component analysis

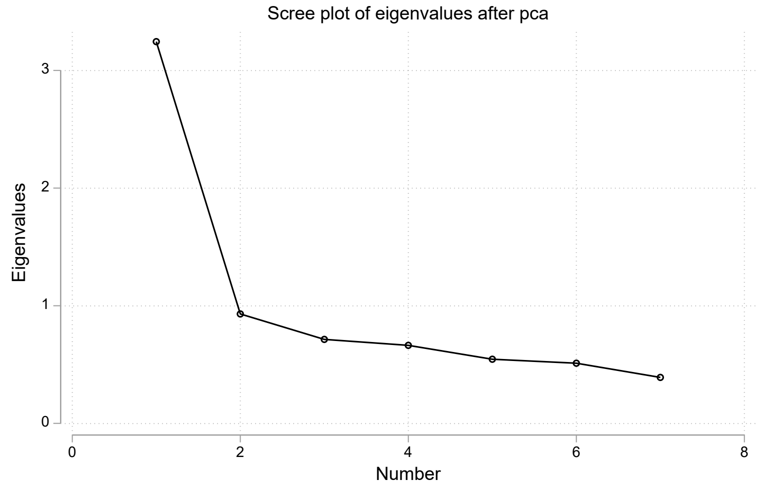

Note. A PCA results in one factor with eigenvalues above 1.0 (3.245 for factor 1)

Plot after parallel analysis

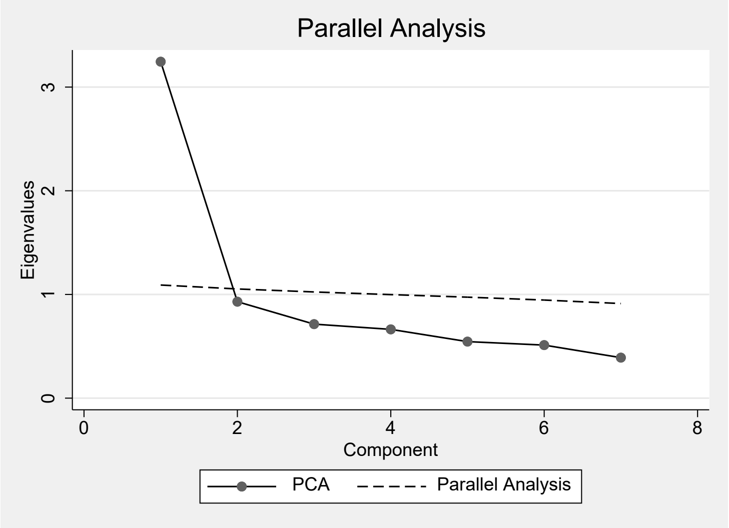

Note. Parallel analysis with randomly generated eigenvalues results in one factor.

Factor analysis (principal factors extraction)

Factor	Eigenvalue	Difference	Proportion	Cumulative
Factor1	2.603	2.418	1.146	1.146
Factor2	0.185	0.204	0.082	1.227
Factor3	-0.018	0.050	-0.008	1.219
Factor4	-0.069	0.056	-0.030	1.189
Factor5	-0.125	0.020	-0.055	1.134
Factor6	-0.145	0.016	-0.064	1.071
Factor7	-0.160	.	-0.070	1.000

Factor loadings (no rotation, since only 1 factor is retained)

Variable	Factor1	Uniqueness
norm_1	0.709	0.498
norm_2	0.550	0.697
norm_3	0.656	0.570
norm_4	0.385	0.852
norm_5	0.722	0.478
norm_8	0.510	0.740
norm_9	0.662	0.561

C3. Exploratory factor analysis perceived disrespect: nine items

Test of appropriateness	
Determinant of the correlation matrix	0.011
Bartlett's test of sphericity	*p*=0.000
KMO	0.945

Determining the number of factors to extract

Screeplot after principal component analysis	Plot after parallel analysis
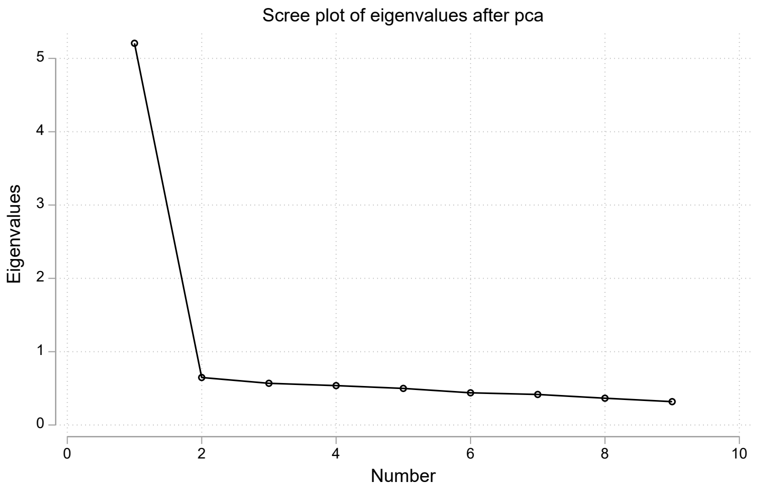	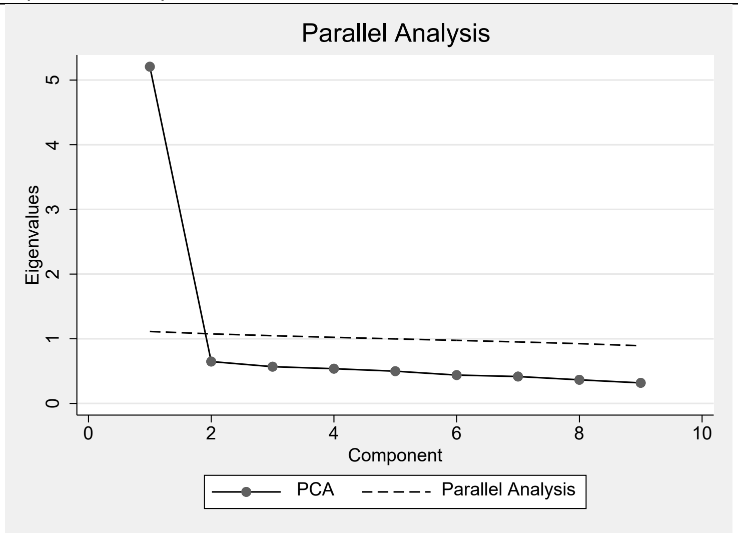
Note. A PCA results in one factors with eigenvalue above 1.0 (5.206 for factor 1)	*Note.* Parallel analysis with randomly generated eigenvalues results in one factor.

Factor analysis (principal factors extraction)

Factor	Eigenvalue	Difference	Proportion	Cumulative
Factor1	4.703	4.573	1.069	1.069
Factor2	0.130	0.090	0.029	1.098
Factor3	0.040	0.050	0.009	1.107
Factor4	-0.010	0.046	-0.002	1.105
Factor5	-0.056	0.030	-0.013	1.092
Factor6	-0.086	0.004	-0.020	1.073
Factor7	-0.090	0.022	-0.020	1.052
Factor8	-0.111	0.008	-0.025	1.027
Factor9	-0.119	.	-0.027	1.000

Factor loadings (no rotation, since only 1 factor is retained)

Variable	Factor1	Uniqueness
percdis_1	0.780	0.391
percdis_2	0.675	0.545
percdis_3	0.759	0.423
percdis_4	0.669	0.552
percdis_5	0.792	0.373
percdis_6	0.658	0.567
percdis_7	0.706	0.502
percdis_8	0.719	0.484
percdis_9	0.735	0.460

Scale reliability

Factor 1 (perceived disrespect item 1, 2, 3, 4, 5, 6, 7, 8, 9)	Cronbach's alpha	0.904

Note: Since we are conducting the main analyses with the composite measure of norm-support with 7 norms (without norm 6 and 7; see above and manuscript pp. 6-7) we, accordingly, created a composite measure for perceived disrespect with the same 7 norms to be used in our main analyses.

D. Average Marginal Effects

D1. The relation between perceived disrespect and thermometer ratings

At norm-support level:	dy/dx	Std.Err.	t	*P>t*	[95%Conf.	Interval]
2 SD below mean	-0.256	0.054	-4.700	*0.000*	-0.362	-0.149
1 SD below mean	-0.253	0.037	-6.890	*0.000*	-0.325	-0.181
Mean	-0.251	0.025	-10.110	*0.000*	-0.299	-0.202
1 SD above mean	-0.248	0.028	-9.020	*0.000*	-0.302	-0.194
2 SD above mean	-0.246	0.042	-5.830	*0.000*	-0.328	-0.163

D2. The relation between perceived disrespect and political trust

At norm-support level:	dy/dx	Std.Err.	t	*P>t*	[95%Conf.	Interval]
2 SD below mean	-0.239	0.055	-4.330	*0.000*	-0.348	-0.131
1 SD below mean	-0.196	0.037	-5.250	*0.000*	-0.270	-0.123
Mean	-0.154	0.025	-6.110	*0.000*	-0.203	-0.104
1 SD above mean	-0.111	0.028	-4.020	*0.000*	-0.165	-0.057
2 SD above mean	-0.068	0.042	-1.620	*0.106*	-0.151	0.015

D3. The relation between perceived disrespect and political talk

At norm-support level:	dy/dx	Std.Err.	t	*P>t*	[95%Conf.	Interval]
2 SD below mean	0.087	0.059	1.490	*0.137*	-0.028	0.202
1 SD below mean	0.105	0.040	2.650	*0.008*	0.027	0.183
Mean	0.123	0.027	4.600	*0.000*	0.071	0.175
1 SD above mean	0.141	0.029	4.810	*0.000*	0.083	0.198
2 SD above mean	0.159	0.045	3.540	*0.000*	0.071	0.246

D4. The relation between perceived disrespect and Information-seeking

At norm-support level:	dy/dx	Std.Err.	t	*P>t*	[95%Conf.	Interval]
2 SD below mean	0.028	0.057	0.490	*0.628*	-0.084	0.140
1 SD below mean	-0.011	0.039	-0.290	*0.774*	-0.087	0.065
Mean	-0.050	0.026	-1.920	*0.055*	-0.101	0.001
1 SD above mean	-0.089	0.029	-3.110	*0.002*	-0.145	-0.033
2 SD above mean	-0.127	0.044	-2.920	*0.004*	-0.213	-0.042

E. Analyses with additional control variables

E1. Variation in norm-support – Extra controls

	Norm-Support		
	b	se	*p*
Age	0.002	0.002	*0.360*
Gender *(ref.=Men)*			
Women	0.038	0.059	*0.519*
Education *(ref.=Higher)*			
Lower	-0.375	0.076	*0.000*
Intermediate	-0.277	0.063	*0.000*
Cynicism	0.201	0.033	*0.000*
Populist attitude	-0.199	0.031	*0.000*
Polarized attitude	-0.160	0.019	*0.000*
Ideology	-0.096	0.012	*0.000*
Region *(ref.=Wallonia)*	0.103	0.057	*0.071*
Extremity (0-5)	0.012	0.018	*0.520*
Satisfaction with democracy (0-10)	-0.129	0.012	*0.000*
Political interest	-0.033	0.014	*0.017*
Freq. exposure political discussions	-0.045	0.019	*0.020*
Intercept	6.485	0.198	*0.000*
R2	0.206		
Adj. R2	0.200		
N	1737		

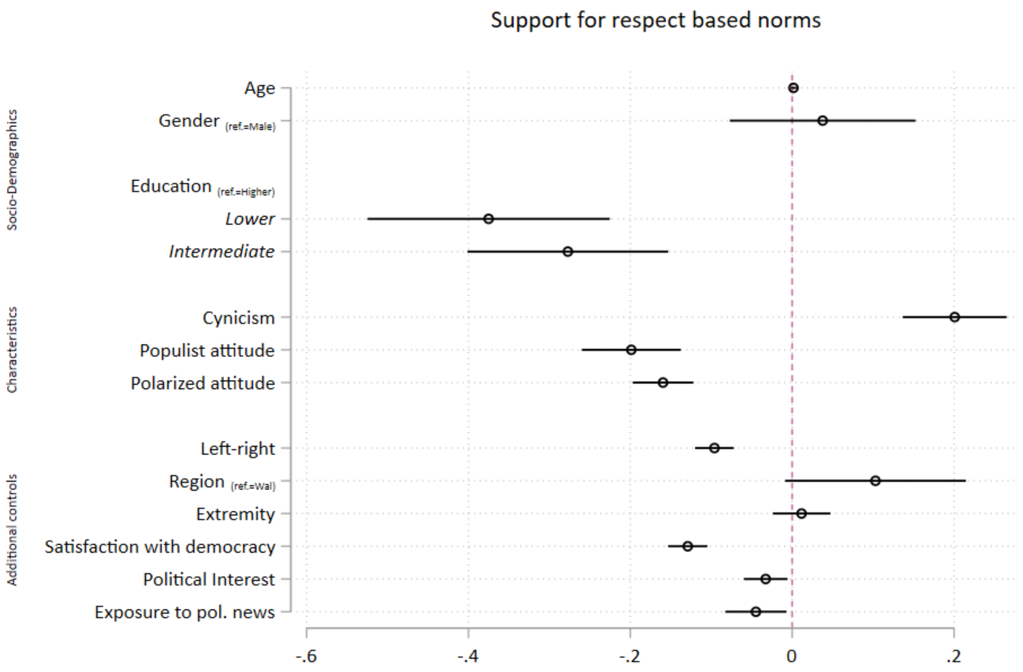

Note. Estimates are the result of an OLS linear regression; Entries are unstandardized coefficients, standard errors and *p-values*

E2. Interactions – Extra controls

	Thermometer (std)			Trust (std)			Political talk (std)			Information-seeking (std)		
	B	se	*p*	b	se	*p*	b	se	*p*	b	se	*p*
Perc. occ. Disrespect	-0.193	0.021	*0.000*	-0.065	0.017	*0.000*	0.073	0.023	*0.001*	-0.093	0.019	*0.000*
Norm-support	-0.214	0.020	*0.000*	-0.125	0.017	*0.000*	0.046	0.022	*0.037*	0.015	0.018	*0.416*
Perc. occ. disrespect * Norm-support	-0.008	0.017	*0.641*	0.021	0.014	*0.150*	0.027	0.019	*0.147*	-0.035	0.015	*0.025*
Age	0.002	0.001	*0.193*	0.000	0.001	*0.838*	-0.007	0.001	*0.000*	0.003	0.001	*0.011*
Gender (ref.=men)	0.095	0.039	*0.014*	0.049	0.032	*0.130*	-0.024	0.042	*0.578*	-0.056	0.035	*0.109*
Education (ref=Intermediate)												
Lower	0.037	0.052	*0.472*	-0.097	0.043	*0.025*	-0.012	0.056	*0.834*	0.040	0.047	*0.395*
Higher	0.017	0.042	*0.685*	-0.038	0.035	*0.278*	-0.017	0.046	*0.713*	0.012	0.038	*0.759*
Ideology (left-right)	-0.008	0.008	*0.359*	-0.013	0.007	*0.065*	0.014	0.009	*0.131*	-0.017	0.007	*0.018*
Region (ref.=Wal.)	0.029	0.037	*0.432*	-0.058	0.031	*0.066*	-0.181	0.041	*0.000*	0.020	0.034	*0.557*
Extremity (0-5)	0.001	0.012	*0.960*	-0.004	0.010	*0.666*	0.032	0.013	*0.017*	0.009	0.011	*0.424*
Satisfaction w/ dem. (0-10)	0.184	0.008	*0.000*	0.270	0.007	*0.000*	-0.027	0.009	*0.003*	0.000	0.007	*0.966*
Political interest	0.063	0.009	*0.000*	0.032	0.008	*0.000*	0.122	0.010	*0.000*	0.169	0.008	*0.000*
Freq. exposure political discussions	0.035	0.013	*0.007*	0.025	0.011	*0.020*	0.206	0.014	*0.000*	0.214	0.012	*0.000*
Intercept	-1.392	0.104	*0.000*	-1.333	0.087	*0.000*	-0.835	0.114	*0.000*	-1.613	0.094	*0.000*
R2	0.463			0.620			0.353			0.534		
Adj. R2	0.458			0.617			0.347			0.530		
N	1580			1587			1592			1592		

Note. Estimates are the result of OLS linear regressions using standardized dependent and independent variables.

E3. Linear predictions and marginal effects – Extra controls

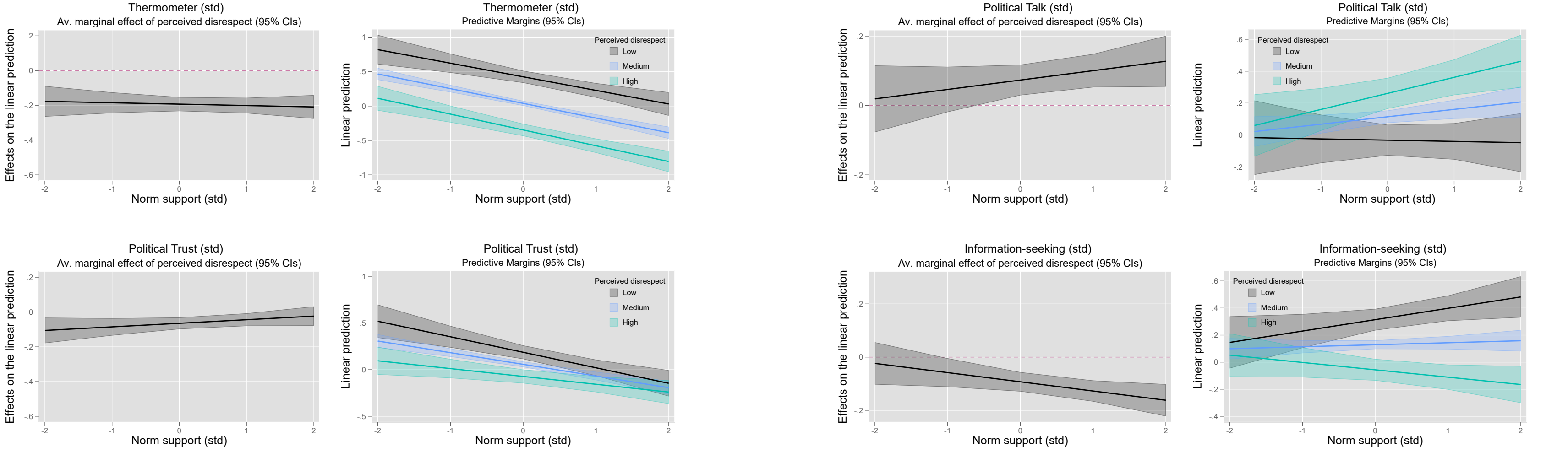

F. Analyses for separate norm-manifestations

This study presented a first step to map variation in support for respect-based norms. To this end, separate manifestations of the broader concept of (dis)respect were grouped together in the main analyses. On the one hand, this allows for a first overall picture of respect in political communication as an overarching norm-construct and including different shades of respect-based norms makes for an encompassing impression. On the other hand, by grouping these separate norm-manifestations, we disregard potential nuances and different patterns for different types of (dis)respect. Therefore, we include the analyses for all separate norm-manifestations here, to provide a complete overview as possible.

Broadly speaking, for RQ1 and H1a–c (variation in norm-support), results for the separate norms present a fairly similar image to the main analyses, yet some differences for the separate norms can be detected. While we overall see fairly similar results for the role of education and cynical, populist an polarized attitudes, for gender and age results differ somewhat more across norms (see Figure F1 and Table F2). For H2a–d (interaction-effects with norm-support), we find some more noteworthy differences from the main analyses across the separate norms, depending on the norm and outcome we study (see Table F3 and Figure F4). In many instances, when looking at the individual norms, the expected interaction-effect appears. For instance, whilst we find no significant interaction-effect of overall norm-support on the relationship between perceived disrespect and affect towards politicians in the main analysis, and contrary to expectations even a positive interaction-effect for political trust, norm-support does have a significant influence in the expected (negative) direction for these outcomes for several separate norms, such as truthfulness and politeness.

F1. Variation in norm-support – Separate norms – Plotted coefficients

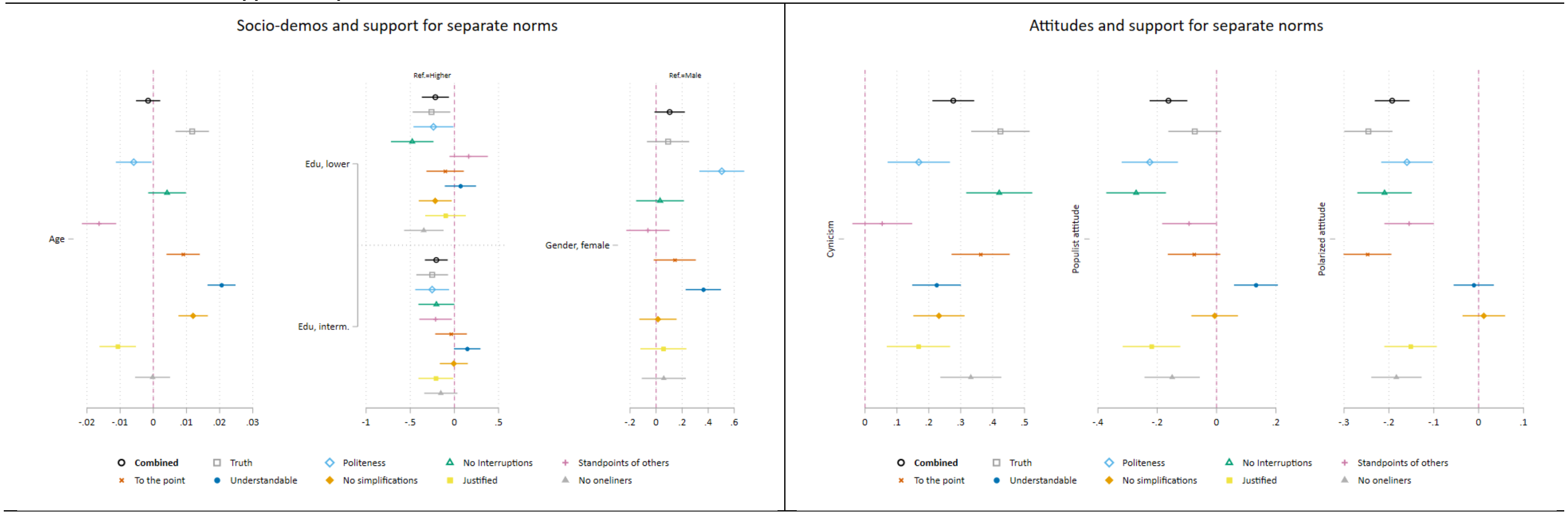

F2. Variation in norm-support – Separate norms

	Norm-support combined			Truthful			Polite			No interruptions			Eng. w/ other standpoints		
	b	se	*p*	b	se	*p*	b	se	*p*	b	se	*p*	b	se	*p*
Age	-0.002	0.002	*0.402*	0.012	0.003	*0.000*	-0.006	0.003	*0.032*	0.004	0.003	*0.152*	-0.016	0.003	*0.000*
Education (ref=int)															
Lower	-0.215	0.078	*0.006*	-0.259	0.109	*0.017*	-0.238	0.116	*0.040*	-0.477	0.123	*0.000*	0.162	0.111	*0.145*
Higher	-0.205	0.066	*0.002*	-0.252	0.092	*0.006*	-0.252	0.098	*0.010*	-0.204	0.104	*0.049*	-0.213	0.094	*0.024*
Gender (ref=male)	0.104	0.059	*0.079*	0.093	0.083	*0.263*	0.505	0.088	*0.000*	0.031	0.094	*0.744*	-0.062	0.085	*0.462*
Cynicism	0.277	0.033	*0.000*	0.424	0.047	*0.000*	0.169	0.050	*0.001*	0.421	0.053	*0.000*	0.054	0.048	*0.259*
Populist attitudes	-0.162	0.032	*0.000*	-0.074	0.045	*0.105*	-0.225	0.048	*0.000*	-0.271	0.051	*0.000*	-0.093	0.046	*0.046*
Polarized attitudes	-0.193	0.020	*0.000*	-0.246	0.027	*0.000*	-0.160	0.029	*0.000*	-0.210	0.031	*0.000*	-0.155	0.028	*0.000*
Ideology *(left-right)*	-0.104	0.013	*0.000*	-0.099	0.018	*0.000*	-0.138	0.019	*0.000*	-0.120	0.020	*0.000*	-0.070	0.018	*0.000*
Region *(ref=Wall.)*	0.087	0.059	*0.141*	-0.061	0.083	*0.459*	0.274	0.088	*0.002*	0.205	0.094	*0.028*	0.628	0.085	*0.000*
Intercept	5.234	0.177	*0.000*	4.510	0.246	*0.000*	5.817	0.262	*0.000*	5.206	0.278	*0.000*	5.297	0.252	*0.000*
R2	0.132			0.137			0.094			0.088			0.070		
Adj. R2	0.128			0.133			0.090			0.084			0.066		
N	1739			1888			1876			1881			1859		

	To-the-point			Understandable			No simplification			Justified			No oneliners		
	b	se	*p*	b	se	*p*	b	se	*p*	b	se	*p*	b	se	*p*
Age	0.009	0.003	*0.000*	0.021	0.002	*0.000*	0.012	0.002	*0.000*	-0.011	0.003	*0.000*	-0.000	0.003	*0.938*
Education (ref=int)															
Lower	-0.105	0.108	*0.332*	0.068	0.090	*0.449*	-0.217	0.095	*0.023*	-0.101	0.117	*0.390*	-0.346	0.114	*0.002*
Higher	-0.038	0.091	*0.678*	0.145	0.077	*0.059*	-0.007	0.081	*0.931*	-0.211	0.100	*0.036*	-0.154	0.096	*0.108*
Gender (ref=male)	0.144	0.082	*0.081*	0.363	0.069	*0.000*	0.015	0.073	*0.842*	0.056	0.090	*0.534*	0.059	0.086	*0.493*
Cynicism	0.362	0.046	*0.000*	0.224	0.039	*0.000*	0.232	0.041	*0.000*	0.167	0.051	*0.001*	0.332	0.049	*0.000*
Populist attitudes	-0.076	0.045	*0.093*	0.133	0.038	*0.000*	-0.006	0.040	*0.875*	-0.219	0.049	*0.000*	-0.150	0.048	*0.002*
Polarized attitudes	-0.248	0.027	*0.000*	-0.011	0.023	*0.636*	0.012	0.024	*0.632*	-0.152	0.030	*0.000*	-0.183	0.029	*0.000*
Ideology *(left-right)*	-0.065	0.018	*0.000*	-0.001	0.015	*0.961*	0.014	0.016	*0.354*	-0.093	0.019	*0.000*	-0.108	0.018	*0.000*
Region *(ref=Wall.)*	-0.065	0.082	*0.432*	-0.058	0.069	*0.402*	0.240	0.073	*0.001*	-0.034	0.090	*0.702*	-0.448	0.086	*0.000*
Intercept	4.431	0.244	*0.000*	2.533	0.205	*0.000*	3.176	0.216	*0.000*	5.773	0.269	*0.000*	5.195	0.257	*0.000*
R2	0.106			0.151			0.068			0.054			0.093		
Adj. R2	0.102			0.147			0.064			0.049			0.089		
N	1886			1861			1860			1860			1859		

Note. Estimates are the result of an OLS linear regression; Entries are unstandardized coefficients, standard errors and *p-values*

F3. Interactions – Separate norms

Truth	Thermometer (std)			Trust (std)			Political talk (std)			Information-seeking (std)		
	b	se	*p*	b	se	*p*	b	se	*p*	b	se	*p*
Perc. untruthful (std)	-0.235	0.022	*0.000*	-0.168	0.022	*0.000*	0.109	0.023	*0.000*	-0.081	0.023	*0.000*
Support truthful (std)	-0.323	0.022	*0.000*	-0.290	0.022	*0.000*	-0.026	0.023	*0.269*	-0.089	0.023	*0.000*
Perception*Support	-0.086	0.022	*0.000*	-0.041	0.023	*0.071*	-0.023	0.024	*0.337*	-0.025	0.023	*0.289*
Age	0.006	0.001	*0.000*	0.005	0.001	*0.001*	-0.002	0.001	*0.189*	0.009	0.001	*0.000*
Education (ref=int)												
Lower	-0.118	0.059	*0.047*	-0.230	0.060	*0.000*	-0.130	0.063	*0.040*	-0.139	0.061	*0.023*
Higher	0.107	0.048	*0.026*	0.213	0.049	*0.000*	0.169	0.051	*0.001*	0.174	0.050	*0.001*
Gender (ref=men)	-0.029	0.044	*0.505*	-0.033	0.044	*0.453*	-0.297	0.047	*0.000*	-0.385	0.045	*0.000*
Ideology (left-right)	0.009	0.009	*0.357*	-0.014	0.009	*0.128*	0.022	0.010	*0.027*	-0.002	0.010	*0.849*
Region (ref=Wal)	-0.041	0.043	*0.336*	0.081	0.043	*0.061*	0.185	0.046	*0.000*	0.000	0.045	*0.997*
Intercept	-0.284	0.097	*0.003*	-0.173	0.098	*0.077*	0.066	0.103	*0.525*	-0.199	0.101	*0.047*
R2	0.195			0.156			0.060			0.092		
Adj. R2	0.191			0.152			0.055			0.088		
N	1804			1812			1817			1816		
**Politeness**	**Thermometer (std)**			**Trust (std)**			**Political talk (std)**			**Information-seeking (std)**		
	b	se	*p*	b	se	*p*	b	se	*p*	b	se	*p*
Perc. impoliteness (std)	-0.111	0.024	*0.000*	-0.051	0.023	*0.029*	0.036	0.023	*0.130*	-0.077	0.023	*0.001*
Support politeness	-0.168	0.024	*0.000*	-0.152	0.024	*0.000*	-0.094	0.024	*0.000*	-0.101	0.023	*0.000*
Perception*Support	-0.074	0.021	*0.001*	-0.059	0.021	*0.005*	0.036	0.021	*0.083*	-0.033	0.020	*0.105*
Age	-0.000	0.001	*0.875*	-0.001	0.001	*0.704*	-0.001	0.001	*0.383*	0.007	0.001	*0.000*
Education (ref=int)												
Lower	-0.099	0.064	*0.125*	-0.223	0.063	*0.000*	-0.135	0.063	*0.032*	-0.120	0.062	*0.051*
Higher	0.115	0.052	*0.028*	0.203	0.051	*0.000*	0.175	0.051	*0.001*	0.176	0.050	*0.000*
Gender (ref=men)	-0.029	0.048	*0.543*	-0.034	0.047	*0.472*	-0.268	0.047	*0.000*	-0.368	0.046	*0.000*
Ideology (left-right)	0.009	0.010	*0.390*	-0.013	0.010	*0.193*	0.017	0.010	*0.085*	-0.007	0.010	*0.458*
Region (ref=Wal)	0.073	0.047	*0.118*	0.167	0.046	*0.000*	0.171	0.046	*0.000*	0.039	0.045	*0.382*
Intercept	-0.057	0.105	*0.587*	0.028	0.104	*0.784*	0.046	0.103	*0.655*	-0.128	0.101	*0.205*
R2	0.059			0.061			0.059			0.090		
Adj. R2	0.054			0.057			0.054			0.086		
N	1803			1811			1816			1816		

Interruptions	Thermometer (std)			Trust (std)			Political talk (std)			Information-seeking (std)		
	b	se	*p*	b	se	*p*	b	se	*p*	b	se	*p*
Perc. interruptions (std)	-0.149	0.023	*0.000*	-0.064	0.023	*0.006*	0.083	0.024	*0.000*	-0.063	0.023	*0.007*
Support no interruptions (std)	-0.254	0.023	*0.000*	-0.216	0.023	*0.000*	-0.008	0.023	*0.739*	-0.057	0.023	*0.013*
Perception*Support	0.030	0.023	*0.195*	0.011	0.023	*0.618*	0.027	0.023	*0.239*	0.028	0.023	*0.224*
Age	0.003	0.001	*0.025*	0.002	0.001	*0.210*	-0.002	0.001	*0.146*	0.008	0.001	*0.000*
Education (ref=int)												
Lower	-0.165	0.063	*0.009*	-0.283	0.063	*0.000*	-0.125	0.063	*0.048*	-0.122	0.062	*0.050*
Higher	0.111	0.051	*0.028*	0.210	0.051	*0.000*	0.179	0.051	*0.000*	0.176	0.050	*0.000*
Gender (ref=men)	-0.040	0.046	*0.386*	-0.041	0.046	*0.376*	-0.311	0.047	*0.000*	-0.399	0.045	*0.000*
Ideology (left-right)	0.004	0.010	*0.680*	-0.015	0.010	*0.141*	0.022	0.010	*0.029*	-0.003	0.010	*0.726*
Region (ref=Wal)	0.050	0.045	*0.271*	0.143	0.045	*0.002*	0.171	0.046	*0.000*	0.021	0.045	*0.636*
Intercept	-0.177	0.102	*0.081*	-0.063	0.101	*0.536*	0.079	0.103	*0.442*	-0.165	0.100	*0.100*
R2	0.095			0.083			0.058			0.084		
Adj. R2	0.091			0.078			0.053			0.079		
N	1813			1821			1826			1825		
**Engaging w/other standpoints**	**Thermometer (std)**			**Trust (std)**			**Political talk (std)**			**Information-seeking (std)**		
	b	se	*p*	b	se	*p*	b	se	*p*	b	se	*p*
Perc. lack of engagement (std)	-0.116	0.024	*0.000*	-0.027	0.024	*0.265*	0.094	0.024	*0.000*	-0.022	0.023	*0.345*
Support engagement (std)	-0.159	0.024	*0.000*	-0.165	0.023	*0.000*	-0.056	0.023	*0.016*	-0.068	0.023	*0.003*
Perception*Support	-0.058	0.020	*0.004*	-0.023	0.020	*0.246*	0.057	0.019	*0.003*	-0.024	0.019	*0.197*
Age	-0.000	0.002	*0.800*	-0.002	0.001	*0.260*	-0.003	0.001	*0.038*	0.007	0.001	*0.000*
Education (ref=int)												
Lower	-0.056	0.065	*0.390*	-0.185	0.064	*0.004*	-0.124	0.064	*0.051*	-0.118	0.062	*0.057*
Higher	0.120	0.053	*0.023*	0.216	0.052	*0.000*	0.158	0.052	*0.002*	0.159	0.050	*0.002*
Gender (ref=men)	-0.073	0.048	*0.126*	-0.079	0.047	*0.093*	-0.292	0.047	*0.000*	-0.388	0.045	*0.000*
Ideology (left-right)	0.019	0.010	*0.060*	-0.005	0.010	*0.600*	0.025	0.010	*0.011*	-0.002	0.010	*0.836*
Region (ref=Wal)	0.073	0.048	*0.124*	0.171	0.047	*0.000*	0.185	0.047	*0.000*	0.035	0.045	*0.436*
Intercept	-0.089	0.107	*0.402*	0.051	0.105	*0.625*	0.115	0.104	*0.269*	-0.093	0.101	*0.361*
R2	0.052			0.059			0.067			0.078		
Adj. R2	0.048			0.054			0.062			0.073		
N	1773			1780			1786			1785		

To the point	Thermometer (std)			Trust (std)			Political talk (std)			Information-seeking (std)		
	b	se	p	b	se	*p*	b	se	*p*	b	se	*p*
Perc. not to the point (std)	-0.184	0.023	*0.000*	-0.099	0.023	*0.000*	0.096	0.024	*0.000*	-0.047	0.023	*0.042*
Support to the point (std)	-0.351	0.022	*0.000*	-0.316	0.022	*0.000*	-0.027	0.023	*0.241*	-0.096	0.023	*0.000*
Perception*Support	-0.060	0.023	*0.010*	-0.035	0.023	*0.125*	0.026	0.024	*0.286*	-0.009	0.024	*0.696*
Age	0.005	0.001	*0.000*	0.004	0.001	*0.007*	-0.002	0.001	*0.172*	0.009	0.001	*0.000*
Education (ref=int)												
Lower	-0.127	0.060	*0.035*	-0.236	0.060	*0.000*	-0.137	0.063	*0.030*	-0.138	0.061	*0.025*
Higher	0.082	0.049	*0.092*	0.176	0.049	*0.000*	0.160	0.051	*0.002*	0.154	0.050	*0.002*
Gender (ref=men)	-0.021	0.044	*0.632*	-0.017	0.044	*0.700*	-0.293	0.047	*0.000*	-0.390	0.045	*0.000*
Ideology (left-right)	0.010	0.009	*0.291*	-0.013	0.009	*0.171*	0.021	0.010	*0.036*	-0.001	0.010	*0.905*
Region (ref=Wal)	-0.041	0.043	*0.344*	0.076	0.044	*0.083*	0.182	0.046	*0.000*	-0.003	0.045	*0.949*
Intercept	-0.260	0.098	*0.008*	-0.128	0.098	*0.193*	0.077	0.103	*0.455*	-0.174	0.100	*0.082*
R2	0.184			0.147			0.057			0.089		
Adj. R2	0.180			0.143			0.053			0.084		
N	1799			1807			1812			1811		

Understandable	Thermometer (std)			Trust (std)			Political talk (std)			Information-seeking (std)		
	b	se	*p*	b	se	*p*	b	se	*p*	b	se	*p*
Perc. not understandable	-0.160	0.026	*0.000*	-0.099	0.025	*0.000*	0.007	0.025	*0.781*	-0.127	0.024	*0.000*
Support understandable	0.002	0.026	*0.938*	0.055	0.026	*0.033*	0.021	0.026	*0.405*	-0.010	0.025	*0.682*
Perception*Support	-0.079	0.020	*0.000*	-0.096	0.019	*0.000*	-0.001	0.019	*0.943*	-0.004	0.018	*0.809*
Age	0.001	0.002	*0.431*	-0.000	0.001	*0.765*	-0.001	0.001	*0.362*	0.008	0.001	*0.000*
Education (ref=int)												
Lower	-0.081	0.065	*0.218*	-0.185	0.064	*0.004*	-0.135	0.064	*0.034*	-0.145	0.062	*0.019*
Higher	0.064	0.053	*0.228*	0.187	0.052	*0.000*	0.183	0.052	*0.000*	0.150	0.050	*0.003*
Gender (ref=men)	-0.051	0.048	*0.296*	-0.068	0.047	*0.150*	-0.295	0.047	*0.000*	-0.394	0.046	*0.000*
Ideology (left-right)	0.026	0.010	*0.010*	0.001	0.010	*0.935*	0.026	0.010	*0.010*	0.005	0.010	*0.620*
Region (ref=Wal)	-0.006	0.047	*0.901*	0.110	0.046	*0.018*	0.175	0.046	*0.000*	0.004	0.045	*0.927*
Intercept	-0.124	0.107	*0.248*	0.030	0.105	*0.776*	0.012	0.105	*0.907*	-0.200	0.102	*0.050*
R2	0.038			0.052			0.051			0.093		
Adj. R2	0.033			0.047			0.046			0.088		
N	1786			1794			1799			1798		

No simplifications	Thermometer (std)			Trust (std)			Political talk (std)			Information-seeking (std)		
	b	se	*p*	b	se	*p*	b	se	*p*	b	se	*p*
Perc. simplifications (std)	-0.111	0.026	*0.000*	-0.078	0.025	*0.002*	0.067	0.025	*0.007*	-0.023	0.024	*0.342*
Support no simplifications (std)	0.069	0.025	*0.006*	0.096	0.024	*0.000*	0.132	0.024	*0.000*	0.138	0.024	*0.000*
Perception*Support	-0.064	0.020	*0.001*	-0.108	0.019	*0.000*	-0.022	0.019	*0.236*	0.021	0.018	*0.257*
Age	0.000	0.002	*0.748*	-0.000	0.001	*0.738*	-0.003	0.001	*0.017*	0.005	0.001	*0.000*
Education (ref=int)												
Lower	-0.083	0.066	*0.208*	-0.190	0.064	*0.003*	-0.127	0.063	*0.045*	-0.112	0.062	*0.069*
Higher	0.077	0.053	*0.149*	0.178	0.052	*0.001*	0.148	0.052	*0.004*	0.154	0.050	*0.002*
Gender (ref=men)	-0.064	0.048	*0.186*	-0.061	0.047	*0.192*	-0.291	0.047	*0.000*	-0.388	0.045	*0.000*
Ideology (left-right)	0.023	0.010	*0.023*	0.001	0.010	*0.958*	0.025	0.010	*0.012*	0.001	0.010	*0.908*
Region (ref=Wal)	-0.033	0.048	*0.497*	0.063	0.047	*0.176*	0.164	0.046	*0.000*	-0.001	0.045	*0.976*
Intercept	-0.059	0.107	*0.582*	0.062	0.104	*0.551*	0.151	0.104	*0.146*	-0.034	0.101	*0.738*
R2	0.026			0.057			0.075			0.087		
Adj. R2	0.021			0.052			0.071			0.082		
N	1764			1770			1775			1774		

Justifications	Thermometer (std)			Trust (std)			Political talk (std)			Information-seeking (std)		
	b	se	*p*	b	se	*p*	b	se	*p*	b	se	*p*
Perc. unjustified (std)	-0.135	0.024	*0.000*	-0.092	0.024	*0.000*	0.084	0.024	*0.000*	0.003	0.023	*0.898*
Support justifications	-0.223	0.024	*0.000*	-0.207	0.023	*0.000*	0.020	0.024	*0.395*	-0.047	0.023	*0.040*
Perception*Support	-0.017	0.021	*0.434*	0.003	0.021	*0.871*	0.020	0.021	*0.341*	-0.027	0.020	*0.182*
Age	0.000	0.001	*0.955*	-0.000	0.001	*0.866*	-0.002	0.001	*0.169*	0.006	0.001	*0.000*
Education (ref=int)												
Lower	-0.057	0.064	*0.379*	-0.178	0.064	*0.005*	-0.119	0.064	*0.065*	-0.106	0.062	*0.091*
Higher	0.118	0.052	*0.024*	0.206	0.052	*0.000*	0.159	0.052	*0.002*	0.187	0.050	*0.000*
Gender (ref=men)	-0.037	0.047	*0.434*	-0.032	0.047	*0.493*	-0.282	0.047	*0.000*	-0.378	0.046	*0.000*
Ideology (left-right)	0.011	0.010	*0.259*	-0.011	0.010	*0.267*	0.025	0.010	*0.015*	0.002	0.010	*0.863*
Region (ref=Wal)	-0.017	0.046	*0.712*	0.095	0.046	*0.041*	0.174	0.047	*0.000*	0.019	0.045	*0.670*
Intercept	-0.042	0.105	*0.689*	0.033	0.104	*0.752*	0.065	0.105	*0.537*	-0.115	0.102	*0.261*
R2	0.071			0.072			0.052			0.074		
Adj. R2	0.066			0.068			0.048			0.069		
N	1765			1772			1777			1776		

**Oneliners**	**Thermometer (std)**			**Trust (std)**			**Political talk (std)**			**Information-seeking (std)**		
	b	se	*p*	b	se	*p*	b	se	*p*	b	se	*p*
Perc. oneliners (std)	-0.174	0.023	*0.000*	-0.072	0.023	*0.002*	0.088	0.024	*0.000*	-0.047	0.023	*0.043*
Support no oneliners (std)	-0.296	0.023	*0.000*	-0.275	0.023	*0.000*	-0.016	0.024	*0.499*	-0.067	0.023	*0.004*
Perception*Support	-0.032	0.022	*0.145*	-0.022	0.022	*0.323*	-0.012	0.023	*0.597*	-0.048	0.022	*0.031*
Age	0.003	0.001	*0.039*	0.001	0.001	*0.327*	-0.001	0.001	*0.308*	0.008	0.001	*0.000*
Education (ref=int)												
Lower	-0.161	0.062	*0.010*	-0.245	0.062	*0.000*	-0.150	0.064	*0.020*	-0.154	0.062	*0.014*
Higher	0.104	0.050	*0.038*	0.213	0.050	*0.000*	0.144	0.052	*0.006*	0.156	0.050	*0.002*
Gender (ref=men)	-0.059	0.046	*0.195*	-0.048	0.046	*0.296*	-0.267	0.047	*0.000*	-0.379	0.046	*0.000*
Ideology (left-right)	0.007	0.010	*0.468*	-0.011	0.010	*0.248*	0.026	0.010	*0.011*	-0.002	0.010	*0.877*
Region (ref=Wal)	-0.093	0.045	*0.040*	0.020	0.045	*0.667*	0.193	0.047	*0.000*	0.001	0.045	*0.974*
Intercept	-0.075	0.100	*0.457*	0.012	0.100	*0.909*	0.029	0.104	*0.777*	-0.111	0.101	*0.272*
R2	0.133			0.111			0.054			0.081		
Adj. R2	0.128			0.107			0.050			0.076		
N	1768			1776			1781			1780		

Note. Estimates are the result of OLS linear regressions using standardized dependent and independent variables.

F4. Linear predictions and marginal effects – Separate norms

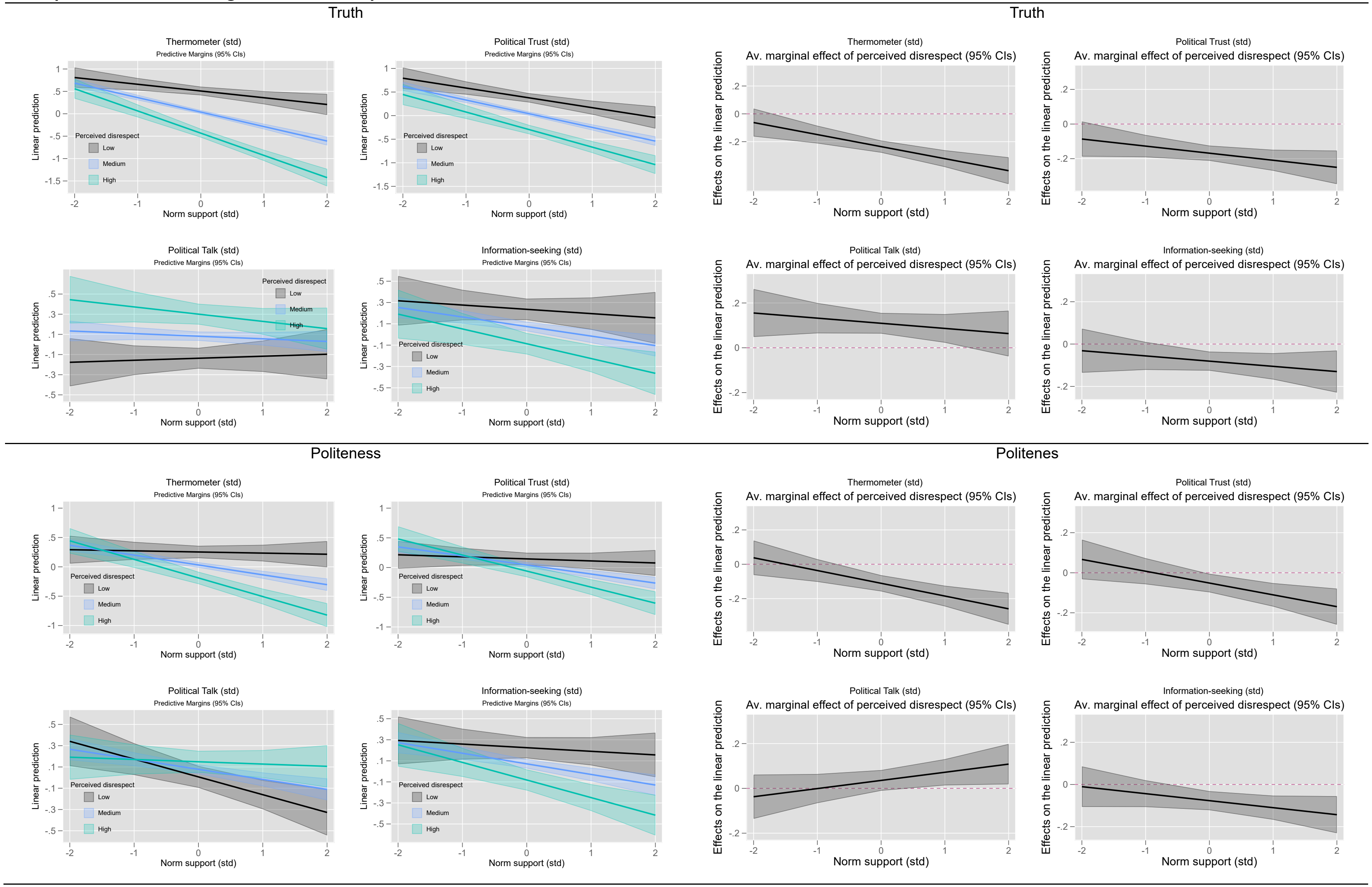

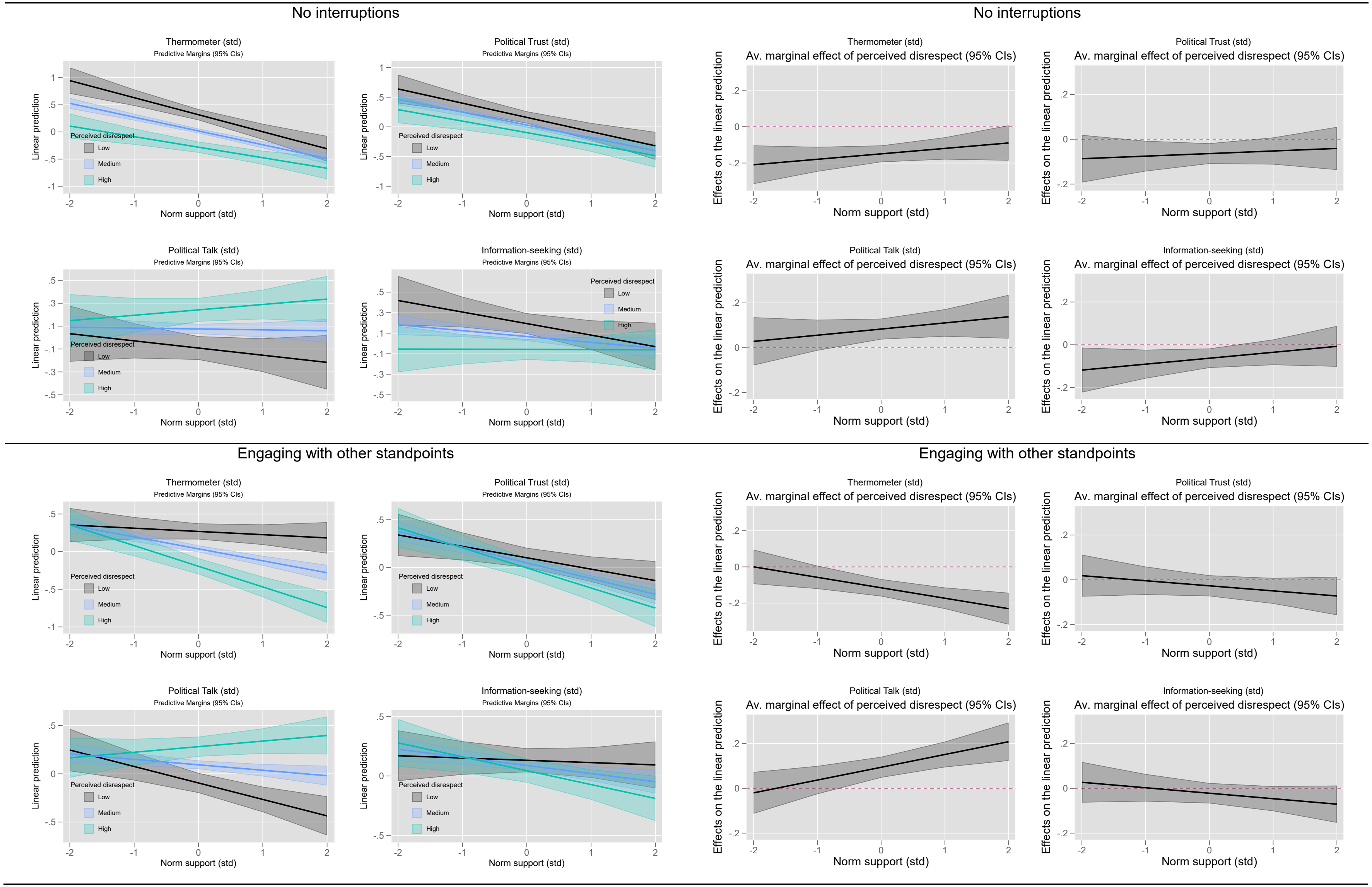
No interruptions
No interruptions
Thermometer (std)
Predictive Margins (95% CIs)
Political Trust (std)
Predictive Margins (95% CIs)
Political Talk (std)
Information-seeking (std)
Av. marginal effect of perceived disrespect (95% CIs)
Linear prediction
Effects on the linear prediction
Norm support (std)
Perceived disrespect
Low
Medium
High
Engaging with other standpoints
Engaging with other standpoints

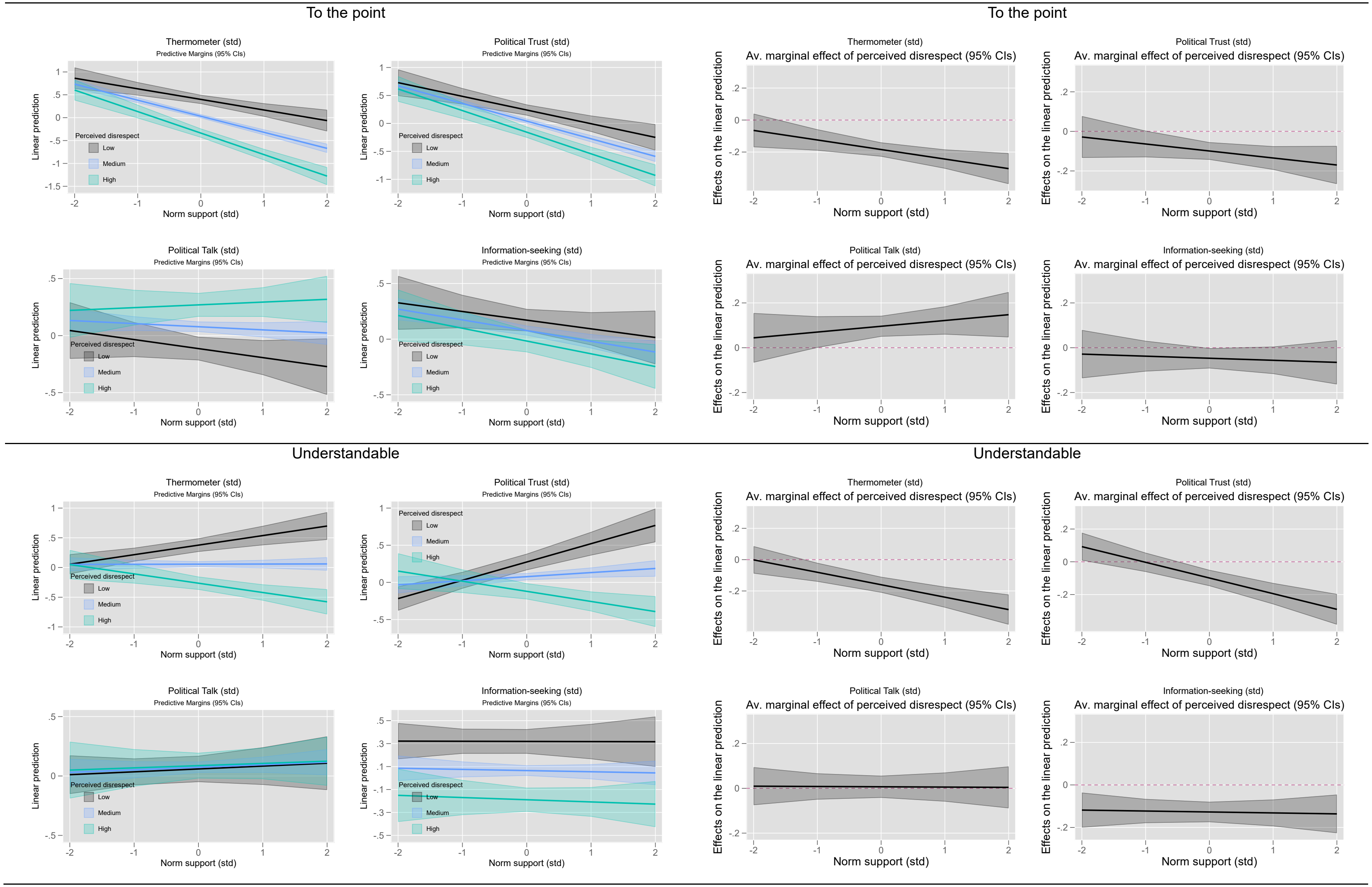

To the point
Thermometer (std)
Predictive Margins (95% CIs)
Linear prediction
Norm support (std)
Perceived disrespect
Low
Medium
High
Political Trust (std)
Predictive Margins (95% CIs)
To the point
Thermometer (std)
Av. marginal effect of perceived disrespect (95% CIs)
Effects on the linear prediction
Norm support (std)
Political Trust (std)
Av. marginal effect of perceived disrespect (95% CIs)
Political Talk (std)
Predictive Margins (95% CIs)
Information-seeking (std)
Predictive Margins (95% CIs)
Political Talk (std)
Av. marginal effect of perceived disrespect (95% CIs)
Information-seeking (std)
Av. marginal effect of perceived disrespect (95% CIs)
Understandable
Thermometer (std)
Predictive Margins (95% CIs)
Political Trust (std)
Predictive Margins (95% CIs)
Understandable
Thermometer (std)
Av. marginal effect of perceived disrespect (95% CIs)
Political Trust (std)
Av. marginal effect of perceived disrespect (95% CIs)
Political Talk (std)
Predictive Margins (95% CIs)
Information-seeking (std)
Predictive Margins (95% CIs)
Political Talk (std)
Av. marginal effect of perceived disrespect (95% CIs)
Information-seeking (std)
Av. marginal effect of perceived disrespect (95% CIs)

No simplifications

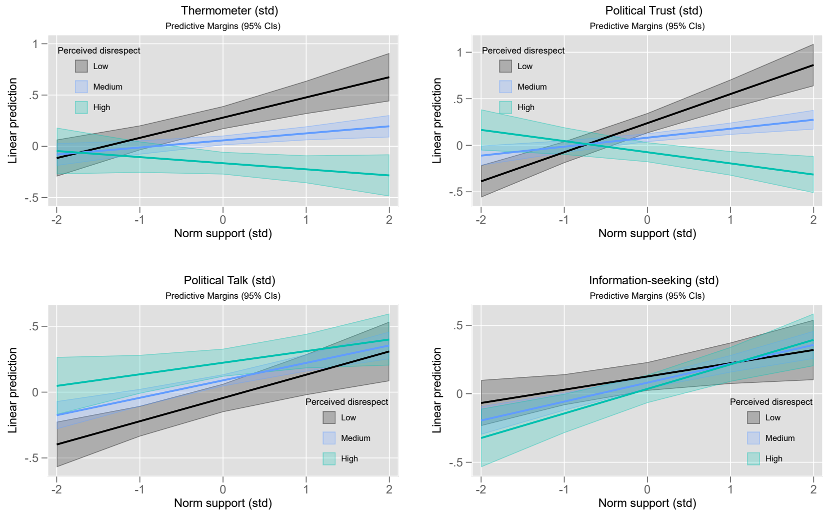

No simplifications

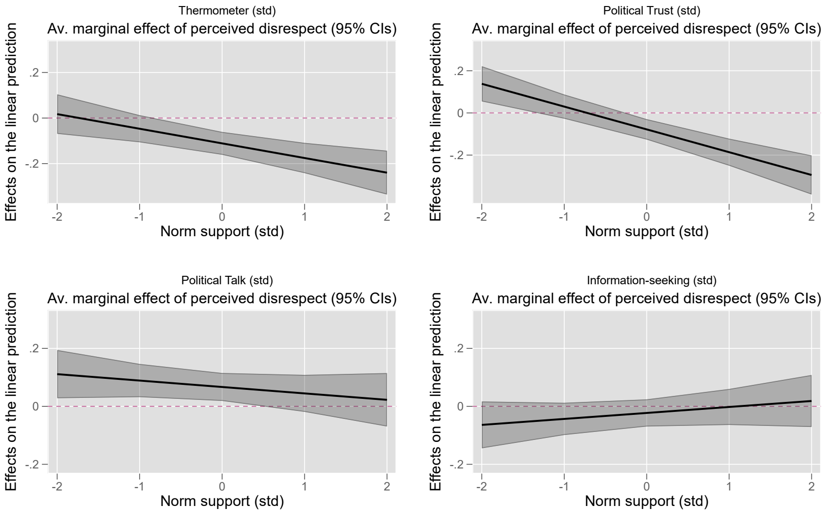

Justifications

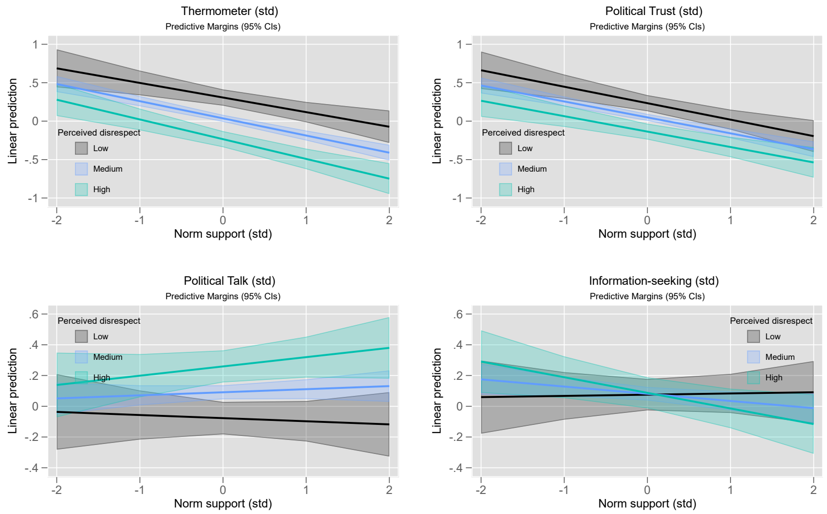

Justifications

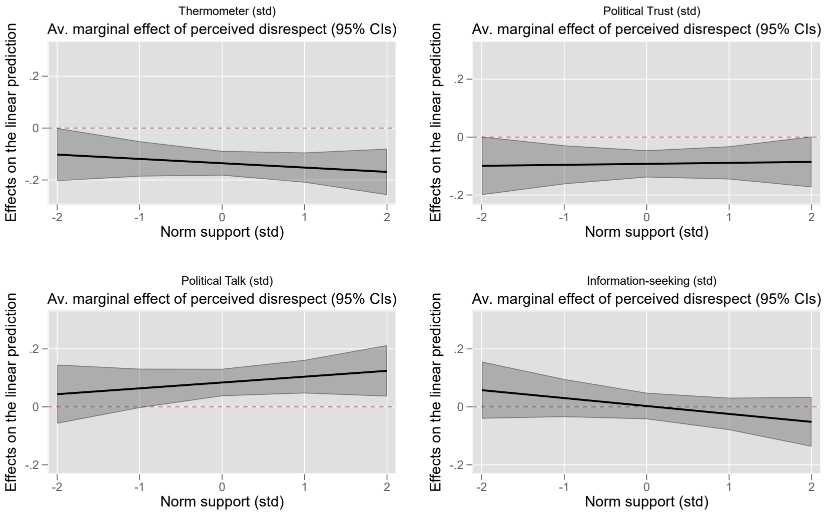

No oneliners

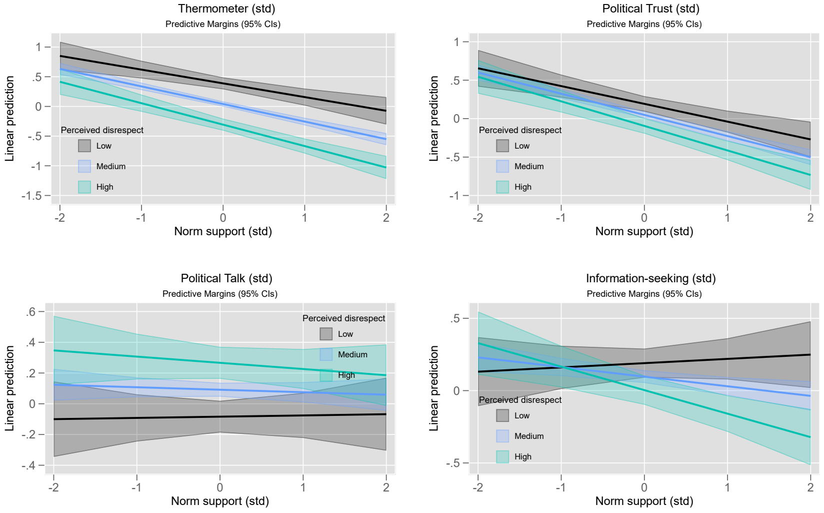

No oneliners

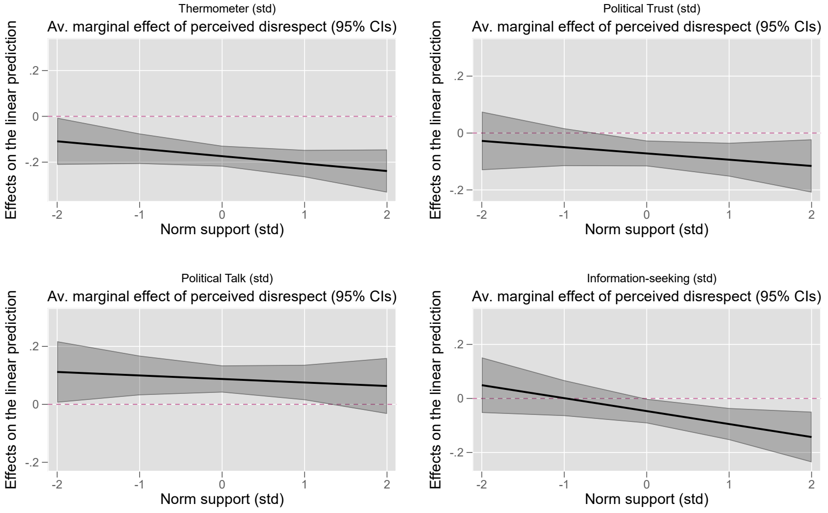

Supplement: nfaf001_Supplementary_Data [file nfaf001_supplementary_data.pdf]
